# Horizontal transfers of *Mariner* transposons between mammals and insects

**DOI:** 10.1186/1759-8753-3-14

**Published:** 2012-09-26

**Authors:** Sarah G Oliveira, Weidong Bao, Cesar Martins, Jerzy Jurka

**Affiliations:** 1 Morphology Department, Bioscience Institute, UNESP - Sao Paulo State University, Botucatu, Sao Paulo, 18618-970, Brazil; 2 Genetic Information Research Institute, 1925 Landings Drive, Mountain View, CA, 94043, USA

**Keywords:** DNA transposon, Genome evolution, Horizontal transfer, *Mariner*

## Abstract

**Background:**

Active transposable elements (TEs) can be passed between genomes of different species by horizontal transfer (HT). This may help them to avoid vertical extinction due to elimination by natural selection or silencing. HT is relatively frequent within eukaryotic taxa, but rare between distant species.

**Findings:**

Closely related *Mariner*-type DNA transposon families, collectively named as *Mariner-1_Tbel* families, are present in the genomes of two ants and two mammalian genomes. Consensus sequences of the four families show pairwise identities greater than 95%. In addition, mammalian *Mariner1_BT* family shows a close evolutionary relationship with some insect *Mariner* families. Mammalian *Mariner1_BT* type sequences are present only in species from three groups including ruminants, tooth whales (*Odontoceti*), and New World leaf-nosed bats (*Phyllostomidae*).

**Conclusions:**

Horizontal transfer accounts for the presence of *Mariner_Tbel* and *Mariner1_BT* families in mammals. *Mariner_Tbel* family was introduced into hedgehog and tree shrew genomes approximately 100 to 69 million years ago (MYA). Most likely, these TE families were transferred from insects to mammals, but details of the transfer remain unknown.

## Findings

In contrast to the vertical transmission of the genetic material from parents to offspring, the horizontal transfer (HT) is a process in which new genetic information is transmitted between different, sometimes distant, species [1,2]. HT is likely to be one of the factors leading to the persistence of transposable elements (TEs) in eukaryotes [3-5], and complicating the evolutionary trees.

The detection of HT is mostly inferential, mainly based on the combination of two types of evidence: unusually high similarity between TE sequences from species that have long diverged from each other, and a limited distribution of one particular TE family within a group of species [6]. To date, numerous HTs have been detected in eukaryotes [6-10], but of particular interest are HTs across distant branches. A recent example of such a rare event is HT of hAT DNA transposon families between vertebrate and invertebrate species [11].

Here, we report two families of *Mariner*-type DNA transposons that have possibly undergone HT from insects to mammals. The first family, called *Mariner_Tbel*, was originally identified in the tree shrew (*Tupaia belangeri*), but families nearly identical to *Mariner_Tbel* were also found in the genome of another mammal, European hedgehog (*Erinaceus europaeus*), and in two ant species: red harvester ant (*Pogonomyrmex barbatus*) and Jerdon's jumping ant (*Harpegnathos saltator*) (Tables 1 and 2). Although the copy numbers and divergence vary between the families, the family consensus sequences reconstructed in each genome show a high level of identity to each other throughout the entire length (approximately 1.3 kb) (Table 1). The lowest identity is found between the two families in *E. europaeus* and ant *P. barbatus* (95.84%), and the highest identity is found between the two ant families (98.45%). Therefore, unless otherwise stated, the four families in the genomes are referred collectively to as *Mariner_Tbel* families. Given the long divergence time between insects and mammals (approximately 1 billion years) [12-14], this high identity strongly indicates that HT took place during the evolutionary history of *Mariner_Tbel* families. This notion is consistent with the fact that mammal *Mariner_Tbel* sequences were found only in two distantly related mammalian species, even though over 30 mammalian genomes were sequenced to date.

**Table 1:** **Pairwise identities (%) between the *Mariner_Tbel *consensus from mammals (*Tupaia belangeri*, *Erinaceus europaeus*) and insects (*Pogonomyrmex barbatus*, *Harpegnathos saltator*)**

	***T. belangeri***	***E. europaeus***	***P. barbatus***
*E. europaeus*	96.55	-	-
*P. barbatus*	98.05	95.84	-
*H. saltator*	97.90	95.69	98.45

**Table 2:** Divergence of Mariner transposable element (TE) families in mammalian and insect genomes

**Family**	**Length (bp)**	**Copy no.**	**Divergence (%)**^**a**^
*Mariner-1_Tbel* (TBel)	1,279	>400	15.0 ± 2 (183)
*Mariner-1_Tbel* (EEr)	1,266	>70	19.4 ± 3 (34)
*Mariner-1_Tbel* (PBa)	1,285	>90	6.3 ± 1 (53)
*Mariner-1_Tbel* (HSa)	1,285	>30	7.2 ± 2 (16)
*TIGGER1* (Tbel)	2,413	>80	21.2 ± 2 (27)
*TIGGER1* (EEu)	2,410	>47	28.0 ± 3 (25)
*TIGGER1* (BT)	2,408	>500	17.3 ± 2 (102)
*TIGGER1* (TTr)	2,419	>580	12.1 ± 1 (159)
*Mariner-28_SIn* (SIn)	1,226	Approximately 14	20.1 ± 3 (12)
*Mariner1_BT* (BT)^b^	1,277	>400	14.7 ± 2 (95)
*Mariner1_BT* (TTr)	1,285	>700	7.6 ± 1 (101)

^a^The divergence represents average pairwise k-distance between individual copies and the consensus. Numbers of individual sequences used for the k-distance calculation are indicated in parentheses.

^b^*Mariner1_BT* families from other *Bos indicus*, *Bos grunniens*, *Bubalus bubalis* are not shown.

**Figure 1:**
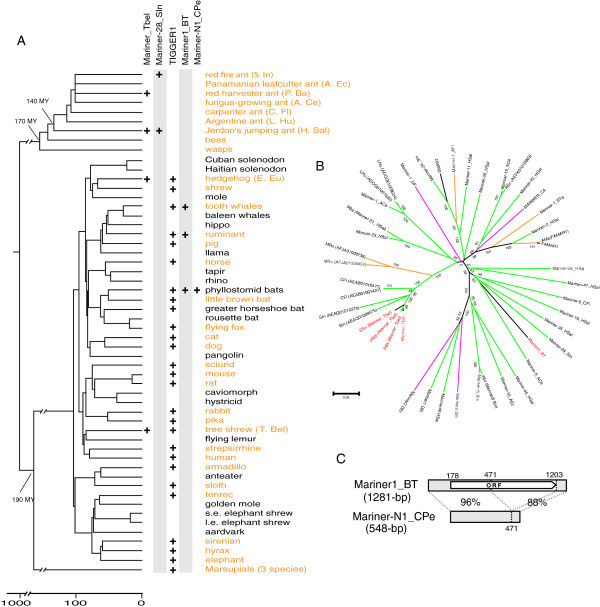
**(A) Distribution of five *Mariner *families (*Mariner_Tbel*, *Mariner1_BT*, *TIGGER1*, *Mariner-28_SIn*, and *Mariner-N1_CPe*) in placental mammals, marsupials, and insects.** The phylogeny is adopted from published trees of insects and mammals [12-15]. Marsupials are represented by three species (*Monodelphis domestica, Macropus eugenii, Sarcophilus harrisii*). The scale bar on the bottom left indicates time of evolutionary branching in the tree. Species with genomic sequences available from public databases are highlighted in orange. (**B**) Phylogenetic position of the two horizontally transferred Mariners (*Mariner_Tbel* and *Mariner1_BT*: red color), relative to other closely related Mariners. Most of the families are from insects with color-coded branches: ants (green), bees (orange), flies (pink) and other insects (blue). The remaining branches (black) are from mammals and planarian (SMAR7). Except for a few individual sequence segments (with accession numbers), all other families are represented by consensus sequences deposited in Repbase (except for highly similar copies). (**C**) Structural relationship between *Mariner1_BT* and *Mariner-N1_CPe* family. The species abbreviations are: ACe (*Atta cephalotes*), AEc (*Acromyrmex echinatior*), AFl (*Apis florea*), AMe (*Apis mellifera*), BTe (*Bombus terrestris*), BT (*Bos taurus*), CA (*Chymomyza amoena*), CFl (*Camponotus floridanus*), Del (*Drosophila elegans*), DEr (*Drosophila erecta*), DF (*Drosophila ficusphila*), EEu (*Erinaceus europaeus*), HSal (*Harpegnathos saltator*), LHu (*Linepithema humile*), MRo (*Megachile rotundata*), PBa (*Pogonomyrmex barbatus*), SIn (*Solenopsis invicta*), SMAR7 (*Schmidtea mediterranea*), Tbel (*Tupaia belangeri*), CPe (*Carollia perspicillata*).

We then estimated the approximate ages of the four *Mariner_Tbel* families in each genome. In mammals, we compared the sequence divergences of *Mariner_Tbel* to an older *Mariner*-type family (*TIGGER1*), relatively common in the mammalian genomes (Table 2). *TIGGER1* elements are present in multiple copies in eutherian mammals, but only one or two degenerated copies were found in marsupial genomes, including *Macropus eugenii*, *Monodelphis domestica*, and *Sarcophilus harrisii* (Figure 1A). Therefore, mammalian *TIGGER1* families likely expanded after the split of marsupials and placentals (190 million years ago (MYA)), but before the placental radiation (approximately 100 MYA) [15]. In the genome of the tree shrew and European hedgehog the divergence of the *TIGGER1* family is 21.2 ± 2% and 28.0 ± 3%, respectively (Table 2). Therefore, based on the divergence of *Mariner_Tbel* in the two mammal genomes (15.0 ± 2% and 19.4 ± 3%, respectively), the ages of *Mariner_Tbel* in the two mammals were calculated to be approximately 134 to 70 MYA and approximately 131 to 69 MYA, respectively. Because it is unlikely that the mammalian *Mariner_Tbel* expanded in the common ancestor of placental mammals before 100 MY, we adjusted the ages to be 100 to 70 MYA and 100 to 69 MYA, respectively (see Figure 1A and [15]).

In the ant genomes, no *Mariner* family was yet identified as unambiguously present in the common ancestor of all ant species. Among potential candidates are the oldest known *Mariner* families present in some of the ant genomes (for example, *Mariner-28_SIn* or *Mariner-94_HSal*; Figure 1). These small families may have expanded in the common ancestor of all ant species (140 MYA) [13], assuming that they were lost in some ant species. Alternatively, these old families might have expanded in some ant species after they split from their common ancestor. Under either scenario, the outermost ages when the two ant *Mariner_Tbel* families expanded could be still estimated by comparing their diversities with the diversity of *Mariner-28_SIn* (Table 2). Based on that, *Mariner_Tbel* family in the red harvester ant (*P. barbatus*) and Jerdon's jumping ant (*H. saltator*) expanded at most approximately 43 and approximately 50 million years ago, respectively.

The above age estimates suggest that the two ant *Mariner_Tbel* families are possibly younger than the mammalian *Mariner_Tbel* families. However, the history of *Mariner_Tbel* can be traced further back in ants and their insect relatives than in mammals. Individual *Mariner_Tbel*-like elements from distinct families, such as AEAQ01009575, AEAB01001421 and AFJA01006902 (Figure 1B), were also found in the genomes of two other ants (*Solenopsis invicta* and *Camponotus floridanus*) as well as in the alfalfa leafcutting bee (*Megachile rotundata*). These *Mariner_Tbel*-like sequences and *Mariner_Tbel* sequences form a single lineage in the phylogenetic tree (Figure 1B), with the bee sequences in a more ancestral position (Figure 1B). The topology of this particular lineage mirrors the evolutionary history of the ant and bee species (Figure 1A). Furthermore, Figure 1B indicates that the *Mariner_Tbel* family and many other similar *Mariner* families in ants and other insects shared a common ancestral sequence. These observations suggest the ancestor of ants *Mariner_Tbel* may have been present in some ant or other insect species very long time ago, probably as far back as the common ancestor of bees and ants (approximately 150 MYA) [14]. Thus, the mammalian *Mariner_Tbel* families probably originated from HTs from insects to mammals through some unknown vectors. Given that the two mammals belong to two distinct lineages, *Mariner_Tbel* in tree shrew and hedgehog may represent two independent HTs (Figure 1A). Notably, we cannot rule out the possibility that the *Mariner_Tbel* families in one of the two ant species, or both, also originated by HTs. This possibility is suggested by two facts: (a) the relatively young ages (at most approximately 43 to 50 MYA) of the two families, (b) the high identity (98.5%) between the two family consensus sequences, even *H. saltator* and *P. barbatus* diverged from each other approximately 100 million years ago [13]. Among insect species, frequent HTs have been documented in flies [16]. Alternatively, *Mariner_Tbel* sequences could have survived for a very long time in either of the two ant genomes before the most recent family expansions.

In addition to mammalian *Mariner_Tbel* families*, Mariner1_BT* DNA families might also have originated by HT from insects (Figure 1A). We were able to obtain high quality consensus of *Mariner1_BT* from bovine species (*Bos taurus, Bos indicus*, *Bos grunniens*, *Bubalus bubalis*) and bottlenosed dolphin *Tursiops truncatus* (Table 2). All the derived consensus sequences show similar lengths (approximately 1,280 bp), and high pairwise identities throughout the entire length (>98%). Blast screening against National Center for Biotechnology Information (NCBI) databases using *Mariner1_BT* consensus sequence as query also detected this family in several other mammalian species, including one bat species, *Carollia perspicillata* (Seba's short-tailed bat), additional ruminants, and whale (Table 3). These BlastN hits show similar score and query coverage (>80% identity to the consensus and >90% coverage). In summary, *Mariner1_BT* type TEs were found only in three taxonomic groups to date: ruminants, tooth whales (*Odontoceti*), and New World leaf-nosed bats (*Phyllostomidae*) (Figure 1A). Notably, in *C. perspicillata* (short-tailed fruit bat) and *Desmodus rotundus* (vampire bat), we also detected a family of non-autonomous DNA transposon, called *Mariner-N1_CPe*, which was likely derived from the bat *Mariner1_BT* family (Figure 1C).

**Table 3:** ***Mariner1_BT *sequences detected in mammals**

**Groups**	**Species**	**Accession**	**Score**	**Query coverage**	**E value**	**Identity**	**Gaps**
Bat	*Carollia perspicillata*	AC152852.2	1,324	98%	0	1,068/1,291 (83%)	87/1,291 (7%)
Ruminants	*Odocoileus hemionus*	AY330343.1	1,278	98%	0	1,061/1,294 (82%)	73/1,294 (6%)
	*Ovis aries*	AC148039.3	1,274	99%	0	1,078/1,309 (82%)	64/1,309 (5%)
	*Muntiacus reevesi*	AC174385.3	1,265	99%	0	1,074/1,312 (82%)	68/1,312 (5%)
	*Muntiacus muntjak vaginalis*	AC152844.3	1,242	99%	0	1,071/1,313 (82%)	71/1,313 (5%)
	*Capra hircus*	EU870890.1	1,142	98%	0	1,031/1,296 (80%)	109/1,296 (8%)
Whale	*Pseudorca crassidens*	AP011081.1	1,833	98%	0	1,180/1,288 (92%)	22/1,288 (2%)

The table shows only top scores using *Mariner*1_BT as BlastN query.

Remarkably, the other closest relatives of *Mariner1_BT* are all found in ant species: *Mariner1_BT* coclusters significantly (bootstrap = 83) with three other ant *Mariner* families (*Mariner-5_ACe*, *Mariner-28_SIn* and *Mariner-35_HSal*) (Figure 1B). Given the vast diversity of Mariners found in insects (Figure 1B), and the confined distribution of *Mariner1_BT* in mammals, we propose *Mariner1_BT* family could also originate from a horizontally transferred insect-like element. Using a similar method above, that is, based on the family divergence and mammalian phylogeny (Table 2 and Figure 1A), we estimated the ages of bovine *Mariner1_BT* to be 90 to 85 MYA, and 90 to 63 MYA for dophin *T. truncatus Mariner1_BT* family. The age of *Mariner1_BT* in bat could not be estimated due to insufficient data. We also could not determine if HT happened in mammals more than once, because the three taxonomic groups that include *Mariner1_BT* are relatively close.

In summary, this is the first report of two cases of horizontally transferred *Mariner* elements (*Mariner_Tbel* and *Mariner1_BT*) between insects and mammals. Previously, four families of DNA transposons from the *hAT* superfamily were also found to be involved in multiple waves of HT between insects and other vertebrates including mammals [11]. This could partially be attributed to the fact that insects are the largest and the most diverse group of invertebrate animals on earth. While insects are the most likely source of the horizontally transferred transposons, the original source or possible intermediaries, such as parasitic insects [11] or viruses, remain unclear. This is complicated by the possibility that recurrent HTs of related *Mariner* elements are likely to take place between different insects [16]. The role of viruses in HT proposed some time ago [17] still remains to be understood. As more genome sequence data become available, more mechanistic details on HT between mammals and insects are likely to emerge.

## Methods

*Mariner* transposable elements from Repbase ( http://www.girinst.org/repbase/) were used as an initial query to screen *Mariners* in diverse genomes available at NCBI (National Center for Biotechnology Information: http://www.ncbi.nlm.nih.gov/). Family consensus sequences were constructed whenever possible. The copy numbers in each family were determined by BLASTN using consensus sequences as queries. Sequence divergence within each family was assessed by the average pairwise k-distance (Kimura two-parameter model) between individual insertions and the corresponding consensus sequences. The k-distance was calculated using the software MEGA 4 [18]. For a given family, individual sequences used in k-distance calculation were randomly chosen from the family members; in most cases individual sequences matched >70% of the consensus length.

We used *Mariner_Tbel* and *Mariner1_BT* as BLASTN queries against Repbase to select top scoring TE entries for phylogeny analysis. Individual sequences selected from GenBank were also used in the tree if Repbase consensus sequences were not available. The sequence alignments are shown in Additional file 1. The alignments were done using the online MAFFT server ( http://mafft.cbrc.jp/alignment/software/). The phylogeny tree was inferred using MEGA 4 [18], using the neighbor joining (NJ) method and k-distances. Branch support was estimated using 1,000 bootstrap replicates.

## Abbreviations

ACe: *Atta cephalotes*; AEc: *Acromyrmex echinatior*; AFl: *Apis florae*; AMe: *Apis mellifera*; BT: *Bos Taurus*; BTe: *Bombus terrestris*; CA: *Chymomyza amoena*; CFl: *Camponotus floridanus*; Del: *Drosophila elegans*; DEr: *Drosophila erecta*; DF: *Drosophila ficusphila*; EEu: *Erinaceus europaeus*; HSal: *Harpegnathos saltator*; HT: Horizontal transfer; LHu: *Linepithema humile*; MRo: *Megachile rotundata*; PBa: *Pogonomyrmex barbatus*; SIn: *Solenopsis invicta*; SMAR7: *Schmidtea mediterranea*; Tbel: *Tupaia belangeri*; TTr: *Tursiops truncatus*.

## Competing interests

The authors declare that they have no competing interests.

## Authors’ contributions

SGO contributed to development of the hypothesis, collection, preparation, analysis and interpretation of data, wrote the first draft of the manuscript, and revised the text. WB contributed to the analysis and interpretation of data, writing and revising the manuscript. CM contributed to the discussion of data and revisions of the manuscript. JJ contributed to development of the hypothesis, interpretation of data and final revisions. All the authors read and approved the final manuscript.

## Supplementary Material

Additional file 1 **Sequence alignments of three *Mariner *families (*Mariner_Tbel*, *Mariner1_BT*, *Mariner-28_SIn*) from eutherian mammals and insects.** Except for a few individual sequence segments (with accession numbers), all other families are represented by consensus sequences deposited in Repbase (excluding highly similar copies). The species are: ACe (*Atta cephalotes*), AEc (*Acromyrmex echinatior*), AFl (*Apis florea*), AMe (*Apis mellifera*), BTe (*Bombus terrestris*), BT (*Bos taurus*), CA (*Chymomyza amoena*), CFl (*Camponotus floridanus*), Del (*Drosophila elegans*), DEr (*Drosophila erecta*), DF (*Drosophila ficusphila*), EEu (*Erinaceus europaeus*), HSal (*Harpegnathos saltator*), LHu (*Linepithema humile*), MRo (*Megachile rotundata*), PBa (*Pogonomyrmex barbatus*), SIn (*Solenopsis invicta*), SMAR7 (*Schmidtea mediterranea*), Tbel (*Tupaia belangeri*). Click here for file

## References

[B1] Syvanen M Horizontal gene transfer: evidence and possible consequences Annu Rev Genet 1994 28 237 261 10.1146/annurev.ge.28.120194.001321 7893125

[B2] Brown JR Ancient horizontal gene transfer Nat Rev Genet 2003 4 121 132 12560809 10.1038/nrg1000

[B3] Hartl DL Lohe AR Lozovskaya ER Modern thoughts on an ancyent marinere: function, evolution, regulation Annu Rev Genet 1997 31 337 358 10.1146/annurev.genet.31.1.337 9442899

[B4] Sanchez-Gracia A Maside X Charlesworth B High rate of horizontal transfer of transposable elements in *Drosophila* Trends Genet 2005 21 200 203 10.1016/j.tig.2005.02.001 15797612

[B5] Schaack S Gilbert C Feschotte C Promiscuous DNA: horizontal transfer of transposable elements and why it matters for eukaryotic evolution Trends Ecol Evol 2010 25 537 546 10.1016/j.tree.2010.06.001 20591532 PMC2940939

[B6] Silva JC Loreto EL Clark JB Factors that affect the horizontal transfer of transposable elements Curr Issues Mol Biol 2004 6 57 71 14632259

[B7] Daniels SB Peterson KR Strausbaugh LD Kidwell MG Chovnick A Evidence for horizontal transmission of the P transposable element between *Drosophila* species Genetics 1990 124 339 355 2155157 10.1093/genetics/124.2.339 PMC1203926

[B8] Loreto EL Valente VL Zaha A Silva JC Kidwell MG *Drosophila mediopunctata* P elements: a new example of horizontal transfer J Hered 2001 92 375 381 10.1093/jhered/92.5.375 11773243

[B9] Maruyama K Hartl DL Evidence for interspecific transfer of the transposable element mariner between *Drosophila* and *Zaprionus* J Mol Evol 1991 33 514 524 10.1007/BF02102804 1664000

[B10] Robertson HM Lampe DJ Recent horizontal transfer of a mariner transposable element among and between *Diptera* and *Neuroptera* Mol Biol Evol 1995 12 850 862 7476131 10.1093/oxfordjournals.molbev.a040262

[B11] Gilbert C Schaack S Pace JK Brindley PJ Feschotte C A role for host-parasite interactions in the horizontal transfer of transposons across phyla Nature 2010 464 1347 1350 10.1038/nature08939 20428170 PMC3004126

[B12] Hedges SB Blair JE Venturi ML Shoe JL A molecular timescale of eukaryote evolution and the rise of complex multicellular life BMC Evol Biol 2004 4 2 10.1186/1471-2148-4-2 15005799 PMC341452

[B13] Moreau CS Bell CD Vila R Archibald SB Pierce NE Phylogeny of the ants: diversification in the age of angiosperms Science 2006 312 101 104 10.1126/science.1124891 16601190

[B14] Brady SG Larkin L Danforth BN Hedges SB, Kumar S Bees, ants, and stinging wasps (Aculeata) In The Timetree of Life 2009 Oxford, UK: Oxford University Press 264 269

[B15] Meredith RW Janečka JE Gatesy J Ryder OA Fisher CA Teeling EC Goodbla A Eizirik E Simão TL Stadler T Rabosky DL Honeycutt RL Flynn JJ Ingram CM Steiner C Williams TL Robinson TJ Burk-Herrick A Westerman M Ayoub NA Springer MS Murphy WJ Impacts of the cretaceous terrestrial revolution and KPg extinction on mammal diversification Science 2011 334 521 524 10.1126/science.1211028 21940861

[B16] Bartolome C Bello X Maside X Widespread evidence for horizontal transfer of transposable elements across *Drosophila* genomes Genome Biol 2009 10 R22 10.1186/gb-2009-10-2-r22 19226459 PMC2688281

[B17] Kidwell MG Lateral transfer in natural populations of eukaryotes Annu Rev Genet 1993 27 235 256 10.1146/annurev.ge.27.120193.001315 8122903

[B18] Tamura K Dudley J Nei M Kumar S MEGA4: Molecular Evolutionary Genetics Analysis (MEGA) software version 4.0 Mol Biol Evol 2007 24 1596 1599 10.1093/molbev/msm092 17488738

