# Supplementary material for: Horizontal transfers of Mariner transposons between mammals and insects
Source: Mob DNA. 2012 Sep 26;3:14. doi: 10.1186/1759-8753-3-14 (PMC3517439; doi:10.1186/1759-8753-3-14)
Supplement: Additional file 1 — Sequence alignments of three Mariner families (Mariner_Tbel, Mariner1_BT, Mariner-28_SIn) from eutherian mammals and insects. Except for a few individual sequence segments (with accession numbers), all other families are represented by consensus sequences deposited in Repbase (excluding highly similar copies). The species are: ACe (Atta cephalotes), AEc (Acromyrmex echinatior), AFl (Apis florea), AMe (Apis mellifera), BTe (Bombus terrestris), BT (Bos taurus), CA (Chymomyza amoena), CFl (Camponotus floridanus), Del (Drosophila elegans), DEr (Drosophila erecta), DF (Drosophila ficusphila), EEu (Erinaceus europaeus), HSal (Harpegnathos saltator), LHu (Linepithema humile), MRo (Megachile rotundata), PBa (Pogonomyrmex barbatus), SIn (Solenopsis invicta), SMAR7 (Schmidtea mediterranea), Tbel (Tupaia belangeri). [file 1759-8753-3-14-S1.pdf]

Additional file 1. Sequence alignment of three *Mariner* families (*Mariner_Tbel, Mariner1-BT, Mariner-28_Sln*) from eutherian mammals and insects.

```
                          1
Mariner-24_SIn            GGTTGTTCGGAAAGTAATTTCGTTTTTTC-CCAACAGAGGG------------------------CAACTTCACACTTAAGCCGCATAATCA
SMAR7                     GGTTGTTCGGAAAGTCATTTCGTTTTTTC-CCAACAGAGGAC--CAATTGTAT-TTTTTCACAGCCAACTTCACACTTAAGCCGCATAATCA
Mariner-11_HSal           GGTTGTTCGGAAAGTAATTTCGGTTTTTT-CAAATAGATGGTGTTAGTTGCCT-TTTTAAGCAGTTAACCTC-ATTCTAAACCAGAAAATCA
Mariner-1_AFl             GATTGTTCGGAAAGTAATTTCGTTTTTT--CACACGAGTGTCGCTAGTTGAAT-GTTTATCCAGCCAACTTC-ATTCTAAACCGTGTTGTTA
Mariner-36_HSal           GGTTGGCAACAAAGTCATGTCGTTTTTCA-CGGATAGATGGCTTTACTTGTCA-TTAAACGCAACTAACTTC-ACTCCAAATCCTATAACCT
Mariner-23_HSal           GGTTGTTCAAAAAGTCATTGCGTTTATTT-CCGATAGATGGCTTTAGTTGTAT-ATTTAATGTATTGAACGC-ATCTATAAATTCATGAGCC
PBa(Mariner-23_HSal)      GGTTGTTCAAAAAGTCATTGCGTTTTTTT-CTGATAGATGGCTTTAGATGTAT-TTGTTATGTATTGAACGC-TTCTATAAATTTATGAGTC
Mariner-42_HSal           GGTTGGCAAGAAAGTAATTTCGGTTTGCG-CCAATAGATAGCGTTATGTTTGT-GTCGTTATTCTTCTGTCA---ATGTTATAATTTTTTTC
Mariner-13_ACe            GGTTGGCAAGAAAGTAATTGCGGTTTTTA-CTGGTAGATGGTGTTGATTGAAG-TTTTTGATATTTTGACGT--TTCGAAGTGTCAAAGTTA
AEc_AEVX01012963          GGTTGGCAAGAAAGTAATTGCGGTTTTTA-CTGGTAGATGGTGTTGATTGAAG-TTTTTGGTATTTTGACGT--TTCGAAGTGTCAAAGT--
Mariner-2_HSal            GGTTGGCAACTAAGTAATTGCGGATTTCA-CTGATAGATGGCGTTAGTTGAAT-TTTTAGGTATATTTACGT-ATTCGAAATGTCAAACCCA
AMe(FAMAR1)               GGTTGGCAACTAAGTAATTGCGGATTTCA-CTCATAGATGGCTTCAGTTGAAT-TTTTAGGTTTGCTGGCGT-AGTCCAAATGTAAAACACA
FAMAR1                    GGTTGGCAACTAAGTACTTGCGGATTTCA-CTCATAGATGGCTTCAGTTGAAT-TTTTAGGTTTGCTGGCGT-AGTCCAAATGTAAAACACA
Mariner-1_BTe             GGTTGGCAACTAAGTGATTGCGGATTTTG-TCAATAGGTGGTCTTAGCACATT-CTTTTGTTTTGTCAACGT-GTTCAAAGCACCTAACCTA
MARINER_CA                GGTTGCCCAAAAAGTAATTGCGGATTTTT-CATATAGTCGGCGTTTATAATTT-TTTTAACAGCTTGTGACT--ATTTAATTGTATTTTTTC
Mariner-1_ACe             GGTTGTTCAAAAAGTCATTGCG-TTTTTT-CCAATAGATGGCGTTGTCCACGT-TTATTTGTATGCGAGCGT-GACATGAATGTCAAATGTT
LHu_ADOQ01001582          GGTTGTTCAAAAAGTCATTGCGTTTTTTT-CCAATAGATAGCGCTGTTCACGT-TTATTTGTATCTGAGCGT-GACATGAATGTCACATGTT
LHu_ADOQ01008024          GGTTGTTCAAAAAGTCATTGCGTTTTTTT-CCAATAGATAGCGCTGTTCACGT-TTATTTGTATCTGAGCGT-GACATGAATGTCACATGTT
Mariner_Tbel              GGTTGTCGGAAAAGTCATGACGCATTTTT-GCAACAGATGGCGCTAGTTCATT-TTTGGAAGTACTAGAGTC-ATTCGCATCTATGTTATTG
HSal(Mariner_Tbel)        GGTTGTCGGAAAAGTCATGACGCACCTTT-GCAACAGATGACGCTAGTTCATT-TTTGGAAGTACTAGAGTC-ATTCGCATCTATGTTATTG
PBa(Mariner_Tbel)         GGTTGTCGGAAAAGTCATGACGCATTTTT-GTAACAGATGGCGCTAGTTCATT-TTTGGAAGTACTAGAGTC-ATTCGCATCTATGTTATTG
EEu(Mariner_Tbel)         GGTTGTCAGAAAAGTCATGATGCATTTTT-GCAACAGATGGCACTAGTTCATT-TTTGGAAGTACTAGAGTC-ATTCACATCTATGTTATTG
CFl_(AEAB01001421)        GGTTGAGTAAAAAGTCATTGCGTATTTTT-GCAACAGATGGCATTAATTCAGT-TTTGGCAATACTAGAGTC-ATTCACACATATGTTTGAC
CFl_(AEAB01018477)        GGTTGAGTAAAAAGTCATTGCGTATTTTT-GCAACAGATGGCATTAATTCAGT-TTTGGCAATACTAGAGTC-ATTCACACATATGTTTGTC
SIn_(AEAQ01009575)        GGTTGTTGGAAAAGTCATAACGTATTTTT-GCAACAAAGGGCTCTAGTTTATT-TTTGGAACTACTAGAGTC-ATTCGCATCTATGTTATTA
SIn_(AEAQ01010279)        AGTTGTC-GGAAAGTCATGACGCATTTTT-GCAACAAAGGGCACTAGGTTATT-TTTGAAAGTACTAGAGTC-ATTTGCGCCTATGTTATTG
MRo_(AFJA01006902)        GGTTATCGGAAAAGTTAGGACCCATTTTTGCAAACAGATGGACCTAATTAAT--TTCTGAGGTACTACACTA---TTTGTTTCCATCTTTTT
MRo_(AFJA01006736)        GGTTGTCGGAAAAGTCATTACGCATTTTTGCAAACAGATGGAGCTAATTAAT--TGCTGAGGTACTACACTC--ATTTTTTTCAATCTTTTT
Mariner-22_HSal           GGTTGGCCAATAAGTCCGTGCGGTTTTTC-TCGATAGATGGCTTCAAGGCGCT-CTGCTAAGCTTTATCTTC-AGTCGAAAGTCTTGAAAGC
Mariner-35_HSal           GGTTGAGTAAAAAGTAATTTCGTATTTTG-CGGGCAGATGGCGTTAAGAGGAA-GTGTTTAGTGCAATCTTC--ACTCAATATCTGACACCA
Mariner1_BT               GGTTGGCCAAAAAGTTCGTTCGGTTTTTT-CCGTAAGATGGCTCTAGTAGCGC-TTAGTTGTCTTTAACTTC-ATTCGAAACAATTTTGTTA
Mariner-5_ACe             GGTTGTCCGGAAAGTTCGTGCCGATTT---TTGGTAGGTGGCGTAAGGACAGG-TCTGAATATATCAGAACGATTGAAAACAAATGACATGC
Mariner-1_DF              GGTTGGCAAGAAAGTCATGTCGTTTTTT--TTAGTAATCGTTTTTAATCTAAT-TTATTTACGACTAATCGA-GCACCAGGGTCACACTTTT
Mariner-6_CFl             GGTTGTCCGGAAAGTTCGTGCGGATTTC--TCAATAGATGTCGTTCAAAGGCGAATTTTACAAGTTTATGTC-GATGTGATATGTCAAATGC
Mariner-16_HSal           GGTCTATGCATAATTCCGTAGCGAAATT--CCGACAGATGTCGTTCGCAGTTG-TTTTTCCGCTCGCACATGTGTCAAACATATGAAGTGTC
Mariner-47_HSal           GGTTGGCAAATAAGTTCGTTCGGTTTTTG-TTTCAGTGTAAGTTTCATGCGGA-TTCGCTTTTCTTTTCTCA--GCGCTATCGTGCAAATCG
Mariner-45_HSal           GGTTGTCTAAAAAGTATTGAGCCTTTTGC-GCACGAGATAGCGTTAGTGTCCC-AAATCTCAGGGTATATGA--GTCGCAGCAGGTCAGGCC
AEc(Mariner-8_SIn)        GGTCATCTAAAAAGTATCCGCGGTTTTTTGTCACTAGATGGTGTTGATGGTC--ATATATCGGGCAACACTA--ATCCGATTGTGTCAAACG
Mariner-8_SIn             GGTCATTTAAAAAGTATCCGCGGTTTTTTGTCACTAGATGGTGTTGATGGTC--ATATATCGGGCAACACTA--ATCCGATTGTATCAAACA
Mariner-16_AEc            GGTGTGGGAAAAAGTTCGTAGCGTTTTTC-CCACTAGATGGCATTAAATGCTC-AGCANTCGGTAACTACTA--ATCGCATCGACAACACTA
Mariner-1_DEl             GGTTGTCAAAAAAGTCTTGCGGTATTT---TCGCTAGTTGGCGCTGAAAGCGC-GTAGTTCTTGTTTTATTC--GTCGCATCAGGTCA----
Mariner-46_HSal           GGTTAAGTAAAAAGTTTTGAGCGTTTT---CCGCTAGATGGCTTTAGTGAGGTCATATCTCACGATGTATAC--GTCGCATCTGGTCATTCT
Mariner-2_DEl             GGTCAAGTAAAAAGTAATCCATTATTTGA-ACAATAGATGACTTTAG----------------------------TCCGATTTGATTTTAGT
Mariner-2_DEr             --TTGGCAAATATCTCCCTTCCGCCTTT--TTGTCTTTTGAATTTCGCGGCTATGTACAAAGCGCTACAGAG--CTCGTATCTGGCAATACT
Mariner-28_SIn            GGTTGG-AAGAAAGTAATGTCGTATTTT--CTCATAGATGGCTTTATGTGTTT-TATTAGAAAGAAACTTCA--ATTTAAATGGCTTAAATG

                         93
Mariner-24_SIn            TTATATATTTTGACAGCTGATATAGCAAGGCTTGCTT---GTGAAAAAGTTCGATTGATTCTACGCAG-TAGTTTTTGTTTGGTGCCCTATC
SMAR7                     TTATATATTTTGACAGCTGATATAGTAAGGTTTGTTT---GTTAAAAATTTTGTTTGATTCTTTGCAA-TAGTTTTTGGAAAAT--------
Mariner-11_HSal           TTTTACATTTTTACAACGCACATTTCAAGCTTCGTTTG--AAAAAAAGTTGTTTTGGAATCTGTCAAG-TTGTTTTTGTTTGGCGTCGCATC
Mariner-1_AFl             TTATATATTTTGAGAGTTGGCATTTCATGGTTTATTTA----AGAAAAATTCGTTTGATTCTGTGCGG-TTGTGTTTTTCGTGTGTGATAAC
Mariner-36_HSal           TTATATCATTTGACAGCTGACATTTCAAGCTTTCTTTA--AAAAAAAAAAGTGCGTCATTCGGTAAAG-AATTGTTTGTTTGGCATTGAGTT
Mariner-23_HSal           CATGCGCGTTTGACAGCTGACACTTATAGCTTCCATC---AAGGAAAAAAGTGCGCGAATCGATCAAC-AATTATTCTTTCTATAACGTTTT
PBa(Mariner-23_HSal)      TATGCGCGTTTAATAGCTGACACTTGTAGGTTCCATCA--AAAAACAAGAGTGCGCAAATCGATCAAC-AATTATTCTTTCTATAGCGTTTT
Mariner-42_HSal           TTTTCAGATTTGGTAGTTGACACTCTTAGGTTACTATA--GAAACAAATTCCGTGTAATTTTGTTTAC-AACTGTTGGTTTACGGTCAATTT
Mariner-13_ACe            TATGTTTGTTTGACAGCTGATACTTTAGTCTTCATTC---AGTAAAAAAATTACATCGATCGAACATT-TAGTTTTTGTTCAGCAATTGTTT
AEc_AEVX01012963          TATGTTTGTTTGGCAGCTGGTACTTTAGTCTTCATTC---AGTAAAAAAATTACATCGATCGAACATT-TAGTTTTTGTTCAGCAATTATTT
Mariner-2_HSal            TATTGTTGTTTGATAGTTGGTACTTCAATTGGCATTC---AGTAAAAAAATTGTATCGATCGGTTGTG-TAGTTTTTGTTTGGCGTCGGTTG
AMe(FAMAR1)               TTTTGTTATTTGATAGTTGGCAATTCAGCTGTCAATC---AGTAAAAAAAGTTTTTTGATCGGTTGCG-TAGTTTTCGTTTGGCGTTCGTTG
FAMAR1                    TTTTGTTATTTGATAGTTGGCAATTCAGCTGTCAATC---AGTAAAAAAAGTTTTTTGATCGGTTTCT-TAGTTTTCGTTTGGCGTTCGTTG
Mariner-1_BTe             TATTGTTTTCTTGTTGTTTGGACTTCCACCTTTAATTT--AGTAAAAAAATTGTATCGATTAGTTATT-TAGTTTCGTTTTTATCCCAATTT
MARINER_CA                TTTTGACATTTATTAGCTGTGACTATTAGCTTGCTTTA--GAAAAAAAGTGCGCGTAATTTTGTTTAC-ATTTGTTTGTTTGGCGCCCTTTT
Mariner-1_ACe             TACGCGCGTTTTGTTGTTGACACTCAACCTAAAAACCA--AAAAAAAAGTTGACGCGATTCGGTCGAG-TTTTGTGCTTGCAATTGTGGTTC
LHu_ADOQ01001582          TATGCGCATTTTGTTGTTGACACTTAACCTAATAACC---AAAAAAAAGTTGACGCAATTCGGTCGAG-TTTTGTGTTTGTAATTGTGGTTT
LHu_ADOQ01008024          TATGCGCATTTTGTTGTTGACACTTAACCTAAAAACC----AAAAAAAGTTGACGCAATTCGGTCGAG-TTTTGTGTTTGTAATTGTGGTTT
Mariner_Tbel              CTATGTCATTTGACAGCTGACAGTTCTAGCTTCATTCT--AAAAAAAA---TAAGCGATTTGGTTGAG-TTTTGGAGATTTT---ACAAGTT
HSal(Mariner_Tbel)        CTATGTCATTTGACAGCTGACAGTTCTAGCTTCATTCT--AAAAAAAAAATTGAGCGATTTGGTTGAG-TTTTGGAGATTTTACAACAAGTT
PBa(Mariner_Tbel)         CTATGTCATTTGACAGCTGACAGTTCTAGCTTCATTCT--AAAAAAAAAATTGAGCGATTTGGTTGAG-TTTTGGAGATTTTATAACAAGTT
EEu(Mariner_Tbel)         CTATGTCATTTGACAGGTGACAGTTCTAGCTTCATTC------AAAAAAATTGAGCAATTTGGTTGAG-TTTTGGAGATTTTACA--AAGTT
CFl_(AEAB01001421)        ATATGCCGTTTAACAGCTGACGGTTTAAGCTTCATTT---GAAAAAAAAATTGAACGATTTAGTTGAGTTTTTGGAGATTTTGCAACAAGTT
CFl_(AEAB01018477)        ATATGCCGTTTAACAGCTGACGGTTTAAGCTTCATTT---GAAAAAAAAATTGAACGATTTAGTTGAGTTTTTGGAGATTTTACAACAAGTT
SIn_(AEAQ01009575)        TTATGTCATTTGACAGTTTACAGTTCTAGTTTTATTCT--AAAAAAAAAATTTAGCGATTTAGTTAA--TTTTGGAGATTTTATAATAAGTT
SIn_(AEAQ01010279)        ACATGTCATTTGACAATTGGCAGTTCTAGTTCCATTCT-AAAAAAAAAAAATGAACAATTTGGTTGAG-TATTGGAAATTTTATAATAAGTT
MRo_(AFJA01006902)        TGTTGTTATGCGATAGTCGGTTGTTCTAGCTTCATTCT-AGAAAAAAAA--------------TACAG-TTTCAGAGATTTTACAGCCAGAG
MRo_(AFJA01006736)        TGTTGCTATGCGATAGTCGGTTGTTCTAGCTTCATTCT-AGAAAAAAAAATTGAGCGATTCGGTACAG-TTTTAAAGATTTTATAGCTAGAG
Mariner-22_HSal           GTATAGTCATTGACAGATGACATTTCAACGTGCATTTA--AAAAATATACGTAAATATTGCTTGTGTA-TCTTGTGTGTTTTGCGTCA-TCT
Mariner-35_HSal           TATATTTTTCTGAAAGGTTAGGTTTTCCTGCGTATTTT--AAGCTGTTATTTGTCAAAATCTATTCAG-TAATTTTTATTTGGCGACATTGT
Mariner1_BT               GATTGTATTGTGACAGCTGTCATATCAGCGTGCATTTC------------TTATCAAAATTGGTGAAT-TTTTGTGTAGCCATTTTAATATT
Mariner-5_ACe             GTACATATCGTAAAAGACGGTACTTCAGGCATACTTG---GAAAAAAATTTGGTTGCGATACGTTAAT-TTTTGTCAGTTTTATACGCCTTT
Mariner-1_DF              TAGTATCGTTTTAAAGCTTATTGTCTTAGGTACTATC---GACGAAAATTTGGTAATTATTCGATAAC-ATTTGTGGAAGCTATAGCAAATT
Mariner-6_CFl             TTTCGTGTTTTGATTAGTGACATTTCAACGCATACTTA--GTGATATTAATTATCCCAATAAATTAAA-AACTTTTTACTTGACATTAGTTT
Mariner-16_HSal           GTACATTAAGTGAAAGCTGAAATTTGTGCCGACATTTT--AAGAAAGTATTCATCGAGATCCGTGACG-TATACGAAGTTTAATTATATTTT
Mariner-47_HSal           AATACACATTTGAAAGGTTATGTTTCACTGTTTATGTT--GTGCTGTGTTTGGTTTGGCTAACATCAG-TATTGTGTGTGCTGCGTAGCTGT
Mariner-45_HSal           TAACGTCATTTGACAGCTGACAGTTGAAGCTAGTAGAC---GTACAAAAAGTGTTGGAATTCTTATAA-TCAGACGTAAGGTATTGCGCCGT
AEc(Mariner-8_SIn)        AACGCGCGTCTGAAAGATGACATTTTAGGTTACATTTT-TTGTAAACAAAGTTTGGTGATTCATCAAT-CGTGTCAAAAGTTATATTGGTTT
Mariner-8_SIn             AACGCGCGTCTGAAAGATGACATTTTAGGTTATATTTT-TTGTAAACAAAGTTTGGTAATTCATCAAT-CGTGTCAAAAGTTATATTGGTTT
Mariner-16_AEc            GACGTGCGTTTAAAAAGTGACATTTCAACCTTTGTTTT--GAGCCGAAAAAGATTGAAATCGGTGAAG-TGGATCAAAAGTTATTTTATTTT
Mariner-1_DEl             ------CCTTTGATTGATTGTCTTT----------------------------------TCTTTTAAG-TCGTTCGTGAGTTATAGCGTCGC
Mariner-46_HSal           ATACTGCGACTTAAAGGTGACATAACGCTTTAAAAAAA-GTTCTTTGACAGTTTTGTGTTTGGTGAAG-CCAGTTGTGTGTTAGAGCG-TTC
Mariner-2_DEl             AGGTGGCGTTAGAAAGTTGAGCTTTCGTACTATAAAAGCTTCTTAGAAAAGTTTTGTGATCGATAACA-CAAACGCTTTGAGAGCTCG--AT
Mariner-2_DEr             ATACATAATTTGAAAGGTCTTGACATAACCTACAAAAC--GACGCTATGCATGATTAGTTTGGATATT-GCGTTCAACAGTTATAGACGTGT
Mariner-28_SIn            CATGCTTTTGTGATAGCTGTAACCTCTAAGCATCATTT-------AGTGTATGTAGAAATTGAATGCA----GAAGCGTTTGTATTCACTTA
```

```
                       185
Mariner-24_SIn         AAATATGGAAAGTAAAAGCAGCATTTT-CGGCATATTTTACTTTTT-TACTATCGAAAAGG-TAAAAATGCTGTTCAAGCAAGA-AAAAAA
SMAR7                  --CGATGGAAAATCGAAAGCAGCATTTT-CGGCATATTTTACTCTTT-TACTATCGCAAAGG-TAAAAATGCAGTTCAAGCAAGG-CGAAAA
Mariner-11_HSal        AAAAATGGAAAACCAACGTGAGCATTTT-CGTCACATTTTGCTTTTT-TATTACCGTAAAGG-CAAAAATGCAGCGCAAGCAAGA-AAAAAA
Mariner-1_AFl          AAAGATGCAAGACCAAAAGGAGCATTTT-CGGCATATTTTACTTTTT-TATTACCGAAAAGA-TAAGAATGCTGTTCAGGCAAGA-AAAAAG
Mariner-36_HSal        AAAAATGGAAAATCAAAACGAGCATTTC-CGCCATATTTTACTTTTT-TACTTCCAAAAAGG-AAAGAACGCTGTGCAAGCCCGT-CAAAAG
Mariner-23_HSal        GGAAATGGAGAGTCAACACGAGCATTTT-CGCCAAGTTTTACTTTAT-TACTTCCGAAAAGG-AAAGAACGCTGTGCAGGCTTGT-GAAAAG
PBa(Mariner-23_HSal)   GAAAATGGAGAATCAACACGAGCATTTT-CGCCAAGTTTTACTTTAT-TACTTCCGAAAAGG-AAAGAACGCTATGCAGGCTTGT-AAAAAG
Mariner-42_HSal        TAACATGGAGTGCAAAAATGATCATTTT-CGACATATTTTACTTTTT-TATTTTCGTAAAGG-CAAGAAAGCGGCTGAGGCTCAC-AAAGAG
Mariner-13_ACe         AAACATGGAGTGTAAAAATGAGCATTTT-CGTCATGTTTTGCTTTTT-TGTTTCCGAAAAGG-AATGAAAGCATCTGAAGCTCAT-AAGGAG
AEc_AEVX01012963       AAACATGGAATGTAAAAATGAACATTTT-CGTCATGTTTTGCTATTT-TGTTTCCGAAAAGG-AATGAAAGCTTCTGAAGCTCAT-AAGGAG
Mariner-2_HSal         AAAAATGGAAAAGCAAAACGAGCATTTT-CGTCATATTTTGCTTTTT-TACTTCCGCAAAGG-AAAAAACGCATCGCAAGCTCAC-AAAAAG
AMe(FAMAR1)            AAAAATGGAAAATCAAAAGGAACATTAT-CGTCATATTTTGCTTTTT-TATTTTCGCAAAGG-GAAAAACGCATCGCAAGCTCAC-AAAAAG
FAMAR1                 AAAAATGGAAAATCAAAAGGAACATTTT-CGTCATATTTTGCTTTTT-TATTTCCGCAAAGG-GAAAAACGCATCGCAAGCTCAC-AAAAAG
Mariner-1_BTe          AAAAATGGAAGAACAAGACGCGCATTTC-AGACATATTTTATTGTAT-TATTTCCGAAAAGG-CAAGAACGCATCGCAAGCACAC-AAGAAG
MARINER_CA             TAATATGGAGTCCACAAAAGAGCATTTC-CGTCATATTTTATATTAT-TATTTCCGTAAAGG-AAAAAACGCAGAGCAGGTTGCT-AAAAAG
Mariner-1_ACe          GAACATGGAGAATTTTGAAGAGCATATT-CGCCATATTCTGTTTTAT-TACTTCAAAAAAGG-GAAGAAAGCAACTGAAGCAAGG-GAAAAG
LHu_ADOQ01001582       AAACATGGAGAATTTTGAAGAGCATATT-CGCCATATTTTGCTTTAT-TACTTTAAAAAAGG-GAAGAAAGCAACTGAAGCATGG-GAAGAG
LHu_ADOQ01008024       AAACATGGAGAATTTTGAAGAGCATATT-CGCCATATTTTGCTTTAT-TACTTTAAAAAAGG-GAAGAAAGCAACTGAAGCATGG-GAAGAG
Mariner_Tbel           GAAGATGGAAGACCAAACTCTGCATTTT-CGCCATATTTTACTGTTT-TACTTCCGTAAAGG-GAAGAACGCGCGTCAAGCTTGT-GAAAAG
HSal(Mariner_Tbel)     GAAGATGGAAGACCAAACTGTGCATTTT-CGCCATATTTTACTGTTT-TACTTCCGTAAAGG-GAAGAACGCGCGTCAAGCTTGT-GAAAAG
PBa(Mariner_Tbel)      GAAGATGGAAGACCAAACTGTGCATTTT-CGCCATATATTACTGTTT-TACTTCCGTAAAGG-GAAGAACGCGCGTCAAGCTTGT-GAAAAG
EEu(Mariner_Tbel)      GAAGATGGAAGACCAAAC--TGCATTTT-CGCCATATTTTACTGTTT-TACTTCCGTAAAGG-GAAGAACATGCATCAAGCTTGT-GAAAAG
CFl_(AEAB01001421)     GAGGATGGAAGGCCAAACTGTGCATTTT-CGTCATATTTTACTGTTT-TATTTCCGTAAAGG-GAAGAACGCGCGTCAAGCTTAT-GAAAAA
CFl_(AEAB01018477)     GAGGATGGAAGGCCAAGCTGTGCATTTT-CGCCATATTTTACTGTTT-TATTTCCGTAAAGG-GAAGAACGCGCGTCAAGCTTAT-GAAAAA
SIn_(AEAQ01009575)     GAAGATGAAAGACCAAACTGTGCATTTT-CGCCATATATTACTGTTTGTACTTCCGTAAAGGAAAAAAACGCGCGTCAGGCTTGTAAAAAAA
SIn_(AEAQ01010279)     GAAGATGGTAAACCAAACTATGTATTTT-CGCCATATATTATTATTT-TTCTTCCATAAA---AAAAAACGCTCGTCAAGCTTGT-GAAAAA
MRo_(AFJA01006902)     AAACATGGAGGACCGCAATGTGCATTTTGCGCCACATTTTATTCTAT-TACTGCTGTAAAGG-GAAAAAGGCACATCAGGTTTGT-GCAAAA
MRo_(AFJA01006736)     AAACATGGAGGACCGCAACGTGCATTTT-CGCCACATTCTACTCTAT-TACTTCCGTAAAGC-GAAGAACGCACGGCAGGTTTGT-GCAAAA
Mariner-22_HSal        GAAAATGGATAACCAGAACGAGCATTTT-CGGCATATTATGCTTTTT-TATTTTAAAAAAGG-CAAAAACGCGGCGCAAACCTGT-AAAAAG
Mariner-35_HSal        AAACATGGAGCAAAACAAGCAGTATTTT-CGGTGTTTGATGCTTTTC-TATTTTCGCAAAGG-GAAAAATGCGACGCAAACCAAAAAAAAAG
Mariner1_BT            GAAGATGGAAGAAAAAAAGCAACATTTT-CGGCATATTATGCTTTAT-TATTTCAAGAAAGG-TAAAAACGCAACTGAAACGCAA-AAAAAG
Mariner-5_ACe          GAATATGGAAGAAAACAAAGTGCATTTT-AGGCATTTAATGCTTTTC-TTTTACCGGAAAGG-CAAAAATGCCACACAAGCGGCA-AACAAG
Mariner-1_DF           AAACATGGAGGACCAAAGTGAGCATTTT-CGGCATATTTTGCTTTTT-TACTTCCGAAAAGG-AAAAAAAGCGGTCGAAGCTCGC-GAAAAA
Mariner-6_CFl          AGAGATGGAAGGAAATAACGTGTTTTTT-CGATGCATAATGCTTTTT-TATTTCCGTAAAGG-AAAGAACGCGACACAAACTCGA-AAAAAA
Mariner-16_HSal        GAACATGGAAGGAAAAAACGTGTATTTT-CGGTGCATTTCGCTTTTT-TATTTCCGAAAAGG-CAAAAACGCGTCGCAAACTCAC-AAAAAG
Mariner-47_HSal        GAACATGGAAATAAATAACGTGCATTAT-CGTCACATTTTGCTCTAT-TATTTTAAAAAAGG-CAAACGAGCTGCGGATGCTCAT-AAAAAG
Mariner-45_HSal        GAAGATGAGTGAACTTTCCGCAAAACTG-CGCTACATTTTACAATTT-TATTTCGATAAAGG-CGCAAATGCTGCGCAGGCCCGT-GAAGAA
AEc(Mariner-8_SIn)     GAAAATGAGTGAAATATCTGAAGAAATA-CGCTATGTGATGCTTTTT-TACTACAAAAAAGG-CAAAAACGCAGCACAAACATGC-AGACAA
Mariner-8_SIn          GAAAATGAGTGAAATTTCTGAAGAAATA-CGTTATGTGATGCTTTTT-TACTACAAAAAAGG-CAAAAACGCAGCACAAACATGC-AGAAAA
Mariner-16_AEc         GAAAATGGAAATAAATAAGGAAAAAATT-CGCTACATTTTGCAGTAT-TACTACGATAAAGG-CAAGAACGCTGCACAGGCTTGT-GAAAAA
Mariner-1_DEl          AATCATGGAGCAAAATAAAGAGAAAATA-CGGCATATTCTACAGTAC-TACTACGATAAAGG-CAAAAATGCATCTCAAGCCGCC-AATAAA
Mariner-46_HSal        CAAAATGGAGGTAAAAAAAGAGAAAATT-CGATACATTCTTCAGTAC-CACTACGACCAAAG-GGAAAAAGCGGAGCAGGCGGCC-AAAAAA
Mariner-2_DEl          AAATATGGAGACCGGCAAAGAAAAAATT-CGCTATATTTTACAATTT-TTCTTTGACAAAGG-CGAAAACGCAAGCCAGGCAGCT-GAAAAT
Mariner-2_DEr          AAACATGGAGTTCACTAACGCCGAAATT-CGCGCTATTTTAAAGTTT-TCCTTCGTTAAAGG-CAAATCCGCTAGAGAAACGTTC-CGTGAG
Mariner-28_SIn         AAATCGAGAGCAAAATAAGTAGTATTTT-CGCTGTTTGATGTTGTTT-TATTTCCGAAAAGG-AAAAAAAACGCGACAC-------AAAAAA

                       277
Mariner-24_SIn         TTATCCGATGTGTATGGAGAAGATGTATTGACAGAAC-GCCAGTGCCAAAACTGGTTTGCAAAATTTCGATCCGGCAATTTTGATGTTGAAG
SMAR7                  TTATGTGATGTGTATGGAGAAGATGTATTGACCGAAC-GACAATGCCAAAATTGGTTTGCAAAATTTCGTTCCGGCAATTTCGATGTTGAAG
Mariner-11_HSal        TTGTGTGCCGTGTATGGAGAGGATGTGTTGACTGAAC-GCCAGTGTCAGAATTGGTTTTCGAAATTTCGTTCTGGGAATTTCGACCTCAAAG
Mariner-1_AFl          TTATGTGAAGTCTATGGAGAAGATGTATTAACAGTAC-GCCAGTGTCAAAATTGGTTTTCAAAATTTCGTTCCGACAATTTCGACATTAAAG
Mariner-36_HSal        TTATGTGCTGTGTATGGTGAGGAATCCTTAACAGAAC-GCCAGTGTCAGCGTTGGTTTGCTCGTTTTCGTTCAGGAGATTTTTCTGTGAAAG
Mariner-23_HSal        TTGCGTAAAGTTTATGGTGACGAAGCCCTAAAAGAAC-GCCAGTGTCAATATTGGTTTGCTCGTTTCCGTTCTGGTGACTACAGCGTGAAAG
PBa(Mariner-23_HSal)   TTACGTAAAATTTATGGTGACGAAGCATTAAAAGAAC-GCCAGTGTCAATATTGGTTTGCTCGTTTCCGTTCTGGTGACTACAGTGTGAAAG
Mariner-42_HSal        ATATGTGAAGTTTATGGTGTTGGTTGCATAACAGAAC-GCACGTGTCAGAATTGGTTTAAAAAATTTCGTTCTGGAGATTTTTCACTTAAAG
Mariner-13_ACe         TTACGTGCTGTGTATGGTGATGAAGCCTTAAAAGAAC-GACAGTGTCAAAATTGGTTTGCCAAATTCCGTTCTGGAGATTTTTCACTCAAAG
AEc_AEVX01012963       TTACGTGCTGTGTATGGTGATGAAGCCTTAAAAGAAC-GACAGTGTCAAAATTGGTTTGCCAAATTCCGTTCTGGAGATTTTTCACTCAAAG
Mariner-2_HSal         TTATGTGCTGTGTATGGTGACGAAGCCTTAAAAGAAC-GGCAGTGTCAAAATTGGTTTGCTAAATTTCGTTCTGGTGATTTTTCACTCAAAG
AMe(FAMAR1)            TTATGTGCTGTTTATGGCGACGAAGCCTTAAAAGAAC-GGCAGTGTCAAAATTGGTTTGACAAATTTCGTTCTGGTGATTTTTCACTCAAAG
FAMAR1                 TTATGTGCTGTTTATGGCGACGAAGCCTTAAAAGAAC-GGCAGTGTCAAAATTGGTTTGCCAAATTTCGTTCTGGTGATTTTTCACTCAAAG
Mariner-1_BTe          TTATGTGCCGTATATGGGAATGAAGCCTTGAAAGAAA-GGCAGTGTCAAAATTGGTTTGCCAAATTTCGTTCTGGTGATTTTTCACTGAAAA
MARINER_CA             TTACGTGATGTGTATGGTGATAAAGCCTTAAAAGAAA-GACAGTGTCAAAATTGGTTTCGCAAATTCCGTTCTGGAGATTTTTCACTTAAAG
Mariner-1_ACe          TTGCGTCGAGTTTACGGCAGAAATGTCGTCAAAAAAC-GCCAGTGCCAGAACTGGTTCGCCCGATTCCGTGATGGTGATTTTTCAGTCAAAG
LHu_ADOQ01001582       TTGCGTCAAGTTTACGGCAGAAATGTCATCGAAAAAT-GTCAGTGCCAGAACTGGTTCGCCCAATTCCGTGGTGGTGATTTTTCAGTTAAAG
LHu_ADOQ01008024       TTGCGTCAAGTTTACGG---AAATGTCATCGAAAAAC-GTCAGTGCCAGAACTGGTTCGCCCGATTCCGTGGTGGTGATTTTTCAGTTAAAG
Mariner_Tbel           TTGCGTAAAGTTTACGGTGACAATGCTCTACAAGAAC-GTCAGTGCCAACGATGGTTCACGAAATTCCGTGCTGGTGATTTTGATCTCAACG
HSal(Mariner_Tbel)     TTGCGTAAAGTTTACGGTGACAATGCTCTACAAGAAC-GTCAGTGCCAACGATGGTTCACGAAATTCCGTGCTGATGATTTTGATCTCAACG
PBa(Mariner_Tbel)      TTGCGTAAAGTTTACGGTGACAATGCTCTACAAGAAC-GTCAGTGCCAACGATGGTTCACGAAATTCCGTGCTGGTGATTTTGACCTCAACG
EEu(Mariner_Tbel)      TTGCGTAAAGTTTATGGTGACAATGCTCTACAAGAAC-GTCAGTGCCAATGATGGTTCATGAAATTCCGTGCTGGTGATTTTGATCTCAACG
CFl_(AEAB01001421)     TTGCGTAAAGTTTACAGTGACGATGCTCTACAAGAAC-GCCAGTGCCAAAACTGTTTCAAGAAATTCCGCGCTGGTGATTTTAATTTCAAAG
CFl_(AEAB01018477)     TTGCGTAAAGTTTACAGTGACGATGCTCTACAAGAAC-GCCAGTGCCAAAACTGTTTCAAGAAATTCCGCGCTGGTGATTTTAATTTCAAAG
SIn_(AEAQ01009575)     TTGCATAAAATTTATGATGACAATGCTGTACAAGAAC-GTCAGTGCCAATGATGGTTCATGAAATTCTGTGCTGGTGATTTTGATCTCAACC
SIn_(AEAQ01010279)     TTGCGTAAAGTTTACGGTGACAATGCTGCACAAGGAC-GTCGGTTCCAATGATGGTTCATGAAAT----TGCTGGTGATTTTGATCTCAACA
MRo_(AFJA01006902)     CTTCAAAAAGTTTACGGTGAAAGTGCTCTTAAAGAAC-GCCAGTGTCAGCGGTGGTTTGAAAAATTTCGTACTGGTGATTTTGACCTCAACG
MRo_(AFJA01006736)     CTACAGAAAGTTTACGGTGAAAGTGCTCTTACAGTAC-GTCAGTGTTAGCGGTAATTTAAAAAATTTCATGCTGGTGACTTTGACCTCAACG
Mariner-22_HSal        ATTTGTGCAATATATGGTGAGGATGCCGTAAAAGAAC-GCGTGTGCCAAAAGTGGTTTGCGAGATTTCGTTGCGGAGATTTTTCCGTCAAAG
Mariner-35_HSal        ATATGTGCTGTGTATGGAGAAGATGCCGTAAGCGAAC-GTGTGTGCCAAAACTGGTTTGCGAAATTTCGTGCTGGTGATACGACATGTCAAG
Mariner1_BT            ATTTGTGCAGTGTATGGAGAAGGTGCTGTGACTGATC-GAACGTGTCAAAAGTGGTTTGCGAAGTTTCGTGCTGGAGATTTCTCGCTGGACG
Mariner-5_ACe          ATATGCGCTGTTTATGGCGAAGGTGCTGTAGCTGAAA-GAACTGTGCGGAAGTGGTTTGCTAGGTTTAAAGCTGGTGATTTCAACCTTGAAG
Mariner-1_DF           TTGTGCGAAGTGTATGGTAAAGATGCCATGAGTGATC-GCCAATGTCAGCGCTGGTTTGCCAAATTCAGGCTTGGAGATTTTGGAGTTAAAG
Mariner-6_CFl          ATATGTGCCGTATACGGGGAAGATGCCGTAAGTGATC-GCATGTGTCAGAAGTGGTTTGCTAAATTTCGTTCAGGAGAAATGAATATTGAAG
Mariner-16_HSal        ATATGCGCCGTGTACGGAGAGAATGCCGTAAGCGAAA-GTGTGTGTCGGAAGTGGTTTGCGAAATTTCGTTGCGGAGATTTTGATCTCCAAG
Mariner-47_HSal        ATATGCCGTGTGTATGGAGATGATGCCTTAACAGAAC-GCGTATGCCAAAAGTGGTATGCCAATTTCCGTTCTGGAGATTTCGACGTCAATG
Mariner-45_HSal        ATTTGTGCTGTTTATGGGCAAGATACGTTATCAAAAG-CAACAGCCAAAAGATGGTTCAGTCGCTTTCGTTCTGGAAATTTCGATGTCCAAG
AEc(Mariner-8_SIn)     ATTTGTGAAGTTTACGGCGCGGATGCTGTAAGTGAAC-GCAGGACGCAGGAGTGGTTTGTTCGATTTCGTTCTGGAAATTTTGATGTCAAAG
Mariner-8_SIn          ATTTGTGAAGTTTACGGCGCTGATGCTGTAAGTGAAC-GCAGGACGCAGGAGTGGTTTGTTCGATTTCGTTCTGGAAATTTTGATGTCAAAG
Mariner-16_AEc         ATTTGTGCTATTTACGGTGAAGATACTCTATCAAAAT-CAGCAGCACGGAAATGGTTCGCTCGTTTTCGTACTGGAAATTTCGATGTGAAAG
Mariner-1_DEl          ATTTGTGCAGTTTATGGACCCGATACAGTTTCCATTT-CCACCGCACAACGATGGTTTCAACGTTTTCGTTCTGGTGTAGAGGTGGTCGAAG
Mariner-46_HSal        ATTTGTGCTGTTTATGGACCCAATACAGTATCGAATG-CAACAGCAAAGCGGTGGTTCCAACGATTCCGTTCTGGTAATATGGACGTCGAAG
Mariner-2_DEl          GTAAATGGAGTATATGGCCCTAATACTGTAACTGCCA-ACCATGCACAATTCTGGTTTCGTCGATTTCGTTCTGGGAATTTTGATGTTAAAG
Mariner-2_DEr          ATTAATGGTGTTTTGGGGGATGGTACTCTATCACTTC-GAACCGCGGAGGAATGGTTTCGACGATTCAGAGCCGGTGAAAACGACACCATGG
Mariner-28_SIn         A---------------GTGACAATGCGATAACTGAACTGCATGTGTCAAAGATGGTTTGCGAAGTTTCGTGCCGGTGATACGACACTCGAAG
```

```
                         369
Mariner-24_SIn        ATGCACCA------------------------------------------------------------------------------------
SMAR7                 ACGCACCA------------------------------------------------------------------------------------
Mariner-11_HSal       ATGCACCA------------------------------------------------------------------------------------
Mariner-1_AFl         ATGCGCCA------------------------------------------------------------------------------------
Mariner-36_HSal       ATGCACCA------------------------------------------------------------------------------------
Mariner-23_HSal       ATGCACCT------------------------------------------------------------------------------------
PBa(Mariner-23_HSal)  ATGCACCT------------------------------------------------------------------------------------
Mariner-42_HSal       ATGACCAA------------------------------------------------------------------------------------
Mariner-13_ACe        ATAATCAA------------------------------------------------------------------------------------
AEc_AEVX01012963      ATAACCAA------------------------------------------------------------------------------------
Mariner-2_HSal        ATGAACAA------------------------------------------------------------------------------------
AMe(FAMAR1)           ACGAAAAA------------------------------------------------------------------------------------
FAMAR1                ATGAAAAA------------------------------------------------------------------------------------
Mariner-1_BTe         ATGCTCAA------------------------------------------------------------------------------------
MARINER_CA            ATGAGCCA------------------------------------------------------------------------------------
Mariner-1_ACe         ACGCTCAT------------------------------------------------------------------------------------
LHu_ADOQ01001582      ACGCTCAT------------------------------------------------------------------------------------
LHu_ADOQ01008024      ACGCTCAT------------------------------------------------------------------------------------
Mariner_Tbel          ACGCTCCT------------------------------------------------------------------------------------
HSal(Mariner_Tbel)    ACGCTCCT------------------------------------------------------------------------------------
PBa(Mariner_Tbel)     ACGCTCCT------------------------------------------------------------------------------------
EEu(Mariner_Tbel)     ATGCTCCT------------------------------------------------------------------------------------
CFl_(AEAB01001421)    ATGCTACT------------------------------------------------------------------------------------
CFl_(AEAB01018477)    ATGCTACT------------------------------------------------------------------------------------
SIn_(AEAQ01009575)    ATTGTCCT------------------------------------------------------------------------------------
SIn_(AEAQ01010279)    ACTCTCCT------------------------------------------------------------------------------------
MRo_(AFJA01006902)    ACACTCCT------------------------------------------------------------------------------------
MRo_(AFJA01006736)    ACACTCCT------------------------------------------------------------------------------------
Mariner-22_HSal       ACAAACCA------------------------------------------------------------------------------------
Mariner-35_HSal       ATGGTGAG------------------------------------------------------------------------------------
Mariner1_BT           ATGCTCCA------------------------------------------------------------------------------------
Mariner-5_ACe         ATCAAGAA------------------------------------------------------------------------------------
Mariner-1_DF          ACGCCCCA------------------------------------------------------------------------------------
Mariner-6_CFl         ATGCTCCT------------------------------------------------------------------------------------
Mariner-16_HSal       ATGCTCCT------------------------------------------------------------------------------------
Mariner-47_HSal       ATGCGCCC------------------------------------------------------------------------------------
Mariner-45_HSal       ATGCTCCT------------------------------------------------------------------------------------
AEc(Mariner-8_SIn)    ATCGACCT------------------------------------------------------------------------------------
Mariner-8_SIn         ATCGACCT------------------------------------------------------------------------------------
Mariner-16_AEc        ATGAACCT------------------------------------------------------------------------------------
Mariner-1_DEl         ATGCGCCA------------------------------------------------------------------------------------
Mariner-46_HSal       ATGAGACA------------------------------------------------------------------------------------
Mariner-2_DEl         ATGAGGCA------------------------------------------------------------------------------------
Mariner-2_DEr         ATAAGCCAGCCGGCGGAAGACCTGTGACGACAAATACCGATCAAATCATGGAATACATCGAGTTAGACCGGCATGTGGCATCTCGTGACATC
Mariner-28_SIn        ATGAGAAG------------------------------------------------------------------------------------

                         461
Mariner-24_SIn        --------------------------------------------------------------------------------------------
SMAR7                 --------------------------------------------------------------------------------------------
Mariner-11_HSal       --------------------------------------------------------------------------------------------
Mariner-1_AFl         --------------------------------------------------------------------------------------------
Mariner-36_HSal       --------------------------------------------------------------------------------------------
Mariner-23_HSal       --------------------------------------------------------------------------------------------
PBa(Mariner-23_HSal)  --------------------------------------------------------------------------------------------
Mariner-42_HSal       --------------------------------------------------------------------------------------------
Mariner-13_ACe        --------------------------------------------------------------------------------------------
AEc_AEVX01012963      --------------------------------------------------------------------------------------------
Mariner-2_HSal        --------------------------------------------------------------------------------------------
AMe(FAMAR1)           --------------------------------------------------------------------------------------------
FAMAR1                --------------------------------------------------------------------------------------------
Mariner-1_BTe         --------------------------------------------------------------------------------------------
MARINER_CA            --------------------------------------------------------------------------------------------
Mariner-1_ACe         --------------------------------------------------------------------------------------------
LHu_ADOQ01001582      --------------------------------------------------------------------------------------------
LHu_ADOQ01008024      --------------------------------------------------------------------------------------------
Mariner_Tbel          --------------------------------------------------------------------------------------------
HSal(Mariner_Tbel)    --------------------------------------------------------------------------------------------
PBa(Mariner_Tbel)     --------------------------------------------------------------------------------------------
EEu(Mariner_Tbel)     --------------------------------------------------------------------------------------------
CFl_(AEAB01001421)    --------------------------------------------------------------------------------------------
CFl_(AEAB01018477)    --------------------------------------------------------------------------------------------
SIn_(AEAQ01009575)    --------------------------------------------------------------------------------------------
SIn_(AEAQ01010279)    --------------------------------------------------------------------------------------------
MRo_(AFJA01006902)    --------------------------------------------------------------------------------------------
MRo_(AFJA01006736)    --------------------------------------------------------------------------------------------
Mariner-22_HSal       --------------------------------------------------------------------------------------------
Mariner-35_HSal       --------------------------------------------------------------------------------------------
Mariner1_BT           --------------------------------------------------------------------------------------------
Mariner-5_ACe         --------------------------------------------------------------------------------------------
Mariner-1_DF          --------------------------------------------------------------------------------------------
Mariner-6_CFl         --------------------------------------------------------------------------------------------
Mariner-16_HSal       --------------------------------------------------------------------------------------------
Mariner-47_HSal       --------------------------------------------------------------------------------------------
Mariner-45_HSal       --------------------------------------------------------------------------------------------
AEc(Mariner-8_SIn)    --------------------------------------------------------------------------------------------
Mariner-8_SIn         --------------------------------------------------------------------------------------------
Mariner-16_AEc        --------------------------------------------------------------------------------------------
Mariner-1_DEl         --------------------------------------------------------------------------------------------
Mariner-46_HSal       --------------------------------------------------------------------------------------------
Mariner-2_DEl         --------------------------------------------------------------------------------------------
Mariner-2_DEr         GCCCAGGAGATGGGAGTTAGTCACCAAACCATTTTAAACCATCTGCAGAAGGCTGGATACAAAAAAAAGCTTGATGTTTGGGTGCCGCATGA
Mariner-28_SIn        --------------------------------------------------------------------------------------------
```

```
                        553
Mariner-24_SIn          --------------------------------------------------------------------------------------------
SMAR7                   --------------------------------------------------------------------------------------------
Mariner-11_HSal         --------------------------------------------------------------------------------------------
Mariner-1_AFl           --------------------------------------------------------------------------------------------
Mariner-36_HSal         --------------------------------------------------------------------------------------------
Mariner-23_HSal         --------------------------------------------------------------------------------------------
PBa(Mariner-23_HSal)    --------------------------------------------------------------------------------------------
Mariner-42_HSal         --------------------------------------------------------------------------------------------
Mariner-13_ACe          --------------------------------------------------------------------------------------------
AEc_AEVX01012963        --------------------------------------------------------------------------------------------
Mariner-2_HSal          --------------------------------------------------------------------------------------------
AMe(FAMAR1)             --------------------------------------------------------------------------------------------
FAMAR1                  --------------------------------------------------------------------------------------------
Mariner-1_BTe           --------------------------------------------------------------------------------------------
MARINER_CA              --------------------------------------------------------------------------------------------
Mariner-1_ACe           --------------------------------------------------------------------------------------------
LHu_ADOQ01001582        --------------------------------------------------------------------------------------------
LHu_ADOQ01008024        --------------------------------------------------------------------------------------------
Mariner_Tbel            --------------------------------------------------------------------------------------------
HSal(Mariner_Tbel)      --------------------------------------------------------------------------------------------
PBa(Mariner_Tbel)       --------------------------------------------------------------------------------------------
EEu(Mariner_Tbel)       --------------------------------------------------------------------------------------------
CFl_(AEAB01001421)      --------------------------------------------------------------------------------------------
CFl_(AEAB01018477)      --------------------------------------------------------------------------------------------
SIn_(AEAQ01009575)      --------------------------------------------------------------------------------------------
SIn_(AEAQ01010279)      --------------------------------------------------------------------------------------------
MRo_(AFJA01006902)      --------------------------------------------------------------------------------------------
MRo_(AFJA01006736)      --------------------------------------------------------------------------------------------
Mariner-22_HSal         --------------------------------------------------------------------------------------------
Mariner-35_HSal         --------------------------------------------------------------------------------------------
Mariner1_BT             --------------------------------------------------------------------------------------------
Mariner-5_ACe           --------------------------------------------------------------------------------------------
Mariner-1_DF            --------------------------------------------------------------------------------------------
Mariner-6_CFl           --------------------------------------------------------------------------------------------
Mariner-16_HSal         --------------------------------------------------------------------------------------------
Mariner-47_HSal         --------------------------------------------------------------------------------------------
Mariner-45_HSal         --------------------------------------------------------------------------------------------
AEc(Mariner-8_SIn)      --------------------------------------------------------------------------------------------
Mariner-8_SIn           --------------------------------------------------------------------------------------------
Mariner-16_AEc          --------------------------------------------------------------------------------------------
Mariner-1_DEl           --------------------------------------------------------------------------------------------
Mariner-46_HSal         --------------------------------------------------------------------------------------------
Mariner-2_DEl           --------------------------------------------------------------------------------------------
Mariner-2_DEr           TTTGACGCAAAAAAACCTTCTGGACCGAATCAACGCCTGCGATATGCTGCTGAAACGGAACGAACTCGACCCATTCTTGAAGCGGATGGTGA
Mariner-28_SIn          --------------------------------------------------------------------------------------------

                        645
Mariner-24_SIn          -----------------------------------------------------------------------------------CGTTCTGGA
SMAR7                   -----------------------------------------------------------------------------------CGTTCTGGA
Mariner-11_HSal         -----------------------------------------------------------------------------------CGTTCTGGA
Mariner-1_AFl           -----------------------------------------------------------------------------------CGTTCTGGA
Mariner-36_HSal         -----------------------------------------------------------------------------------CGGTCAGGC
Mariner-23_HSal         -----------------------------------------------------------------------------------CGCTCAGGT
PBa(Mariner-23_HSal)    -----------------------------------------------------------------------------------CGCTCAGAT
Mariner-42_HSal         -----------------------------------------------------------------------------------CGTTCTGGC
Mariner-13_ACe          -----------------------------------------------------------------------------------CGTTCCGGT
AEc_AEVX01012963        -----------------------------------------------------------------------------------CGTTCCGGT
Mariner-2_HSal          -----------------------------------------------------------------------------------CGCTCTGGT
AMe(FAMAR1)             -----------------------------------------------------------------------------------CGCTCTGGT
FAMAR1                  -----------------------------------------------------------------------------------CGCTCTGGT
Mariner-1_BTe           -----------------------------------------------------------------------------------CGATCTGGC
MARINER_CA              -----------------------------------------------------------------------------------CGTTCAGGT
Mariner-1_ACe           -----------------------------------------------------------------------------------CGCTCCGGT
LHu_ADOQ01001582        -----------------------------------------------------------------------------------CGCTCCGGT
LHu_ADOQ01008024        -----------------------------------------------------------------------------------CGCTCCGGT
Mariner_Tbel            -----------------------------------------------------------------------------------CGGTCAGGG
HSal(Mariner_Tbel)      -----------------------------------------------------------------------------------CGGTCAGGG
PBa(Mariner_Tbel)       -----------------------------------------------------------------------------------CGGTCAGGG
EEu(Mariner_Tbel)       -----------------------------------------------------------------------------------CGGTCAGGG
CFl_(AEAB01001421)      -----------------------------------------------------------------------------------CGGTCAGGA
CFl_(AEAB01018477)      -----------------------------------------------------------------------------------CGGTCAGGA
SIn_(AEAQ01009575)      -----------------------------------------------------------------------------------CGGTCAAGA
SIn_(AEAQ01010279)      -----------------------------------------------------------------------------------CGATCAGGA
MRo_(AFJA01006902)      -----------------------------------------------------------------------------------CGGTCAGGG
MRo_(AFJA01006736)      -----------------------------------------------------------------------------------CGGTCAGGG
Mariner-22_HSal         -----------------------------------------------------------------------------------CGCTTAGGC
Mariner-35_HSal         -----------------------------------------------------------------------------------CGCTCAGGT
Mariner1_BT             -----------------------------------------------------------------------------------CGGTCGGGT
Mariner-5_ACe           -----------------------------------------------------------------------------------CGCCCGGGT
Mariner-1_DF            -----------------------------------------------------------------------------------CGTTCTGGT
Mariner-6_CFl           -----------------------------------------------------------------------------------CGCTCTGGT
Mariner-16_HSal         -----------------------------------------------------------------------------------CGCTCTGGT
Mariner-47_HSal         -----------------------------------------------------------------------------------CGCTCCGGT
Mariner-45_HSal         -----------------------------------------------------------------------------------CGCAGTGGG
AEc(Mariner-8_SIn)      -----------------------------------------------------------------------------------CGCTCTGGT
Mariner-8_SIn           -----------------------------------------------------------------------------------CGCTCTGGT
Mariner-16_AEc          -----------------------------------------------------------------------------------CGCTCCGGT
Mariner-1_DEl           -----------------------------------------------------------------------------------CGCTCCGGA
Mariner-46_HSal         -----------------------------------------------------------------------------------CGCTCTGGT
Mariner-2_DEl           -----------------------------------------------------------------------------------CGCTGCGGA
Mariner-2_DEr           CTGGCGACGAAAAATGGATCACATACGACAATATCAAGCGAAAACGGTCGTGGTCGAAGGCCGGTGAATCGTCCCAAACAGTGGCCGGCGGA
Mariner-28_SIn          -----------------------------------------------------------------------------------CGCACTGGT
```

```
                    737
Mariner-24_SIn        AGGCCAGTTGAAGCTGATGAAGACACAATAAAGGCATTAATTGATGCAAA-CCGGCGAATAACAACTC-GTGAGATCGCTGAGAGGTTAGGT
SMAR7                 AGGCCCGTTGAAGCCGATGAAGACAAAATAAAGGCATTGATAGATGCAAA-CCGCCGAATAACAACTC-GTGAGATTGCTGAGAGGTTAAAT
Mariner-11_HSal       AGGCCAGTTGAGGCTGATGAAGACCAAATAAAGGCATTGGTGGACGCAAA-TCGTCATTTAACAACAC-GAGAAATTGCTGAGAGATTGAAT
Mariner-1_AFl         AGACCAGTTGAAGCTGATGAAGACAAAATAAAGGCACTGATTGAAGCAAA-CCGACGAATAACAACTC-GAGAAATTGCTACGAGATTGAAT
Mariner-36_HSal       CGGCCAATTGAGGTCGATGACGACAAAATAAAAGCATTGCTTGAATCCAA-CCGCCATTATACGACAC-GGGAGATCGCTGAGAAGCTAAAC
Mariner-23_HSal       CGGCCATCGGAGGTTGATGATGATAAAATAAAGGCATTAGTTGAAGCTAA-TCGACGTTCTACCATTC-GTGAGCTCGCTGAGGCACTAAAA
PBa(Mariner-23_HSal)  CGGTCATTGGAGGTTGATAATGAGAAAATAAAGGCATTAGTTGAAGCTAA-TAGACGTTCTACCATTC-GTGAGCTCGCTGAGGCACTAAAA
Mariner-42_HSal       CGACCTTCTGAAGTTGATGAAGACAAAATGAAAGCCATAATTGAATCAAA-TCGTCATATAACTGTGC-GAGAGATTGCAAAAAGGTTAAAT
Mariner-13_ACe        CGACCTATTGATGTTGATGATGACCAAATCAAAGCCATAATTGAATCGAA-TCGTCATATAAGTGTGC-GGGAGATAGCAGAGAGGTTAAAT
AEc_AEVX01012963      CGACCTATTGATGTTGATGATGACCAAATCAAAGCCATAATTGAATCGGA-TCGTCATATAAGTGTGC-GGGAGATAGCAGAGAGGTTAAAT
Mariner-2_HSal        CGTCCAGGTGAAGTTGATGACGACCAAATCAAAGCAATAATCGATGCGGA-TCGTCATAGCACAACAC-GTGAGATTGCAGAGAAGCTCGAT
AMe(FAMAR1)           CGTCCAGTTGAAGTTGATGACGACCTAATCAAAGCAATAATCGATTCGGA-TCGTCACAGTACAACTC-GTGAGATTGCAGAGAAGCTTCAT
FAMAR1                CGTCCAGTTGAAGTTGATGACGACCTAATCAAAGCAATAATCGATTCGGA-TCGTCACAGTACAACAC-GTGAGATTGCAGAGAAGCTTCAT
Mariner-1_BTe         CGTCCAGTTGAAGTTGATGAGACCCATATCAAGGCCATTATCGATTCAGA-TCGTCATAGCACAACGC-GTGAGATTGCAGAGAAGCTCGAT
MARINER_CA            CGGCCAAATGAAGTTGATGATGACCAAATCAAAGCATTAATCGAATTGGA-TCGTCATGTAACTGAGC-GTGAGATAGGAGAGAAGTTAAAT
Mariner-1_ACe         AGGCCTTCCAAGATTGACGACGATGAAATGAAAGCATTGGTGCAAGCAAA-TAAGCATTCAACGGTTC-GGGAGCTTGCTACCGCTCTAAAA
LHu_ADOQ01001582      AGGCCTTCTGAGATTGACGACAATAAAATCAAGGCATTGGTGCAAGCAAA-TAGGCATTCAACGGTTC-GGGAGCTTGCTACCGCTCTAAAA
LHu_ADOQ01008024      AGACCTTCTGAGATTGACGACGATAAAATCAAGGCATTGGTGCAAGCAAA-TAGGCATTCAACAGTTC-GGGAGCTTGCTACCGCTCTAAAA
Mariner_Tbel          AGGCCCACCGAGGTTGATGACGACAAAATAAAGGCATTGATCGAGTCAAA-CCCACGTTACACGACAC-GAGAGATTGCAGAAACATTGAAC
HSal(Mariner_Tbel)    AGGCCCACCGAGGTTGATGACGACAAAATAAAGGCATTGATCGAGTCAAA-CCCACGTTACACGACAC-GAGAGATTGCAGAAACATTAAAC
PBa(Mariner_Tbel)     AGGCCCACCGAGGTTGATGACGACAAAATAAAGGCATTGATCGAGTCAAA-CCCACGTTACACGACAC-GAGAGATTGCAGAAACATTGAAC
EEu(Mariner_Tbel)     AGGCCCACCGAGGTTGATGACGACAAAATAAAGGCATTGATCGAGTCAAA-CCCACGTTACATGACAC-GAGAGATTGCAGAAACATTGAAC
CFl_(AEAB01001421)    AGACCCACCGAGGTTGATGACGACAAAATAAAGGCATTGATCAAGTCAAA-CCCACGTTACACGACAC-GAGAGATTGCAGAAACATTGAAG
CFl_(AEAB01018477)    AGACCCACCGAGGTTGATGACGACAAAATAAAGGCATTGATCAAGTCAAA-CCCACGTTACACGACAC-GAGAGATTGCAGAAACATTGAAG
SIn_(AEAQ01009575)    AGGTCCATCGAAGTTGATGACGACAA----AAGGCATTGATCGAGTCAAA-TCCACGTTACACGACAC-AAGAAATTGCAAGAATATTGAAC
SIn_(AEAQ01010279)    AGATCCATTGAGGTTGATGACGACTAAATGAAGGCATTGATTTAGTCAAA-CCCAGGTTATATGACAC-AAAAGATTACAAAATAATGAAC
MRo_(AFJA01006902)    AGGCCCATTGAAGTTGATGACGATCAAATCAAGGCATTGATCGAATGAAA-TAACCGTTACACTACAC-GGGAAATTGCCGAAATGTTAGAC
MRo_(AFJA01006736)    AGGCCCATTGAAGTCGATGACGATTAAATCAAGGCATTGATCGAATCAAA-TAACCATTACACTATAC-GGGAAATTGCCGAAATGTTAGAC
Mariner-22_HSal       CGACCAAATGAGATCGACAGCGACAAATTGAAGGCGGTAATCGACACCAA-TCTACACTACACGACGC-GGGAGATTGCAGACGTTCTCCAA
Mariner-35_HSal       AGGCCATTGGTGGTTGATGACGACCAAATCAAGACATTGATTGAAAATAACCCCACGTTATACGACAC-GAGAGATCGCAGAGATAATCAAC
Mariner1_BT           AGACCAGTTGAAGTTGATAGCGATCAAATCGAGACATTAATTGAGAACAA-TCAACGTTATACCACGC-GGGAGATAGCCGACATACTCAAA
Mariner-5_ACe         AGGCCCTCCACCACAGATGAAGATCAGATTAAAACACTGATCGAGAATAA-CCCACGCTATACGACAC-GTAAATTAGCAGAAATGTTGAAT
Mariner-1_DF          CGACCATCGGCCGTTTTTGACGAACAAATTCAGGCTTTGCTGGATGAAAA-CCGGCGTTATTCAACGC-GGGAGATGTCAGAGAAGCTCAGT
Mariner-6_CFl         CGGCCAGTCACCACTGATGTCGACCAAATAACAGCTTTAATCGATTCCGA-GCGGCAGATCACAGTTC-GGGAAATCGCAGCGAGGTTAAAC
Mariner-16_HSal       CGGCCAGTCACTACTGATGTCGATCAAATCAAGGCTTTAATCGACTCCGA-CCGGCATTTGACGACTA-CAGAGATCGGACATAACCTCAAC
Mariner-47_HSal       CGCCCGACCGAGATTAATTCAAGTGACATTAAGGCTATAATCGAAGTAAA-TCCATCCCAATCGGTAC-GAGAGATTGCTACGACATTGAAT
Mariner-45_HSal       CGACCAATCACAGAAAAAGTGGATGATATTCTGGCAAAAGTCGAGCAAGA-CCGGCATGTAAGCAGTC-ATGACATCGCCAATGACCTAAAC
AEc(Mariner-8_SIn)    CGGCCAGTCACTGAAAAAGTCGATGAAATTTTGCAACTGGTCAAGCAAGA-CCGGCATGTGAGCTGTC-AGGAGATAGCCAATGCACTAAGA
Mariner-8_SIn         CGGCCAGTCACTGAAAAAGTCGATGAAATTTTGCAACTGGTCGAGCAAGA-CCGGCATGCAAGCTGTC-AGGACATAGCCAATGCACTAAGA
Mariner-16_AEc        CGACCAATCACTGAAAAGTCCGATGAAATCATGGAAAAAGTCGAGCGTGA-TAAGCATGTGAGCACTG-TGGAGATTGCCAGGGAACTAGGC
Mariner-1_DEl         AGGCCTGTCGTCGAAAATTGCGATAAAATCGCTGAATTGGTCGAAAGAGA-CCGGCATAGTAGCAGCC-GTAGCATCGGTCAAGAGCTGGGC
Mariner-46_HSal       AGGCCAATCGTCGAAAATGTCGATAAAATCATGGAAATCGTCGAGTCGGA-CCGGCATGCGAGCACTT-ATTCCATTGCCCAGGAACTGAAG
Mariner-2_DEl         AGGCCAATCGTCAAAAATGCCGACAAAATAATGGAAATCGTTGAGTCTGA-CCGTCATTCAAGCAGTA-AATCAATTGCCCAGGAGCTAAAC
Mariner-2_DEr         AGACCTGTGACGACAAATACCGATCAAATCATGGAATACATCGAGTTAGA-CCGGCATGTGGCATCTC-GTGACATCGCCCAGGAGATGGGA
Mariner-28_SIn        CGATCGACTGTCGCTGATGACGATTAAA----GACTTTAATCGCGAATAA-TCAGCGGTACACGACGCGGAGAGATTGCAGAGATACTCCAT

                    829
Mariner-24_SIn        TTATCGAATTCGACCGTTCATGATCATTTGAAACGCCT-AGGTTTA-ATCTCAAA----------------GCTCGACATATGGGTTCCTCA
SMAR7                 TTGTCGAATTCGACTGTTCATGATCACGTGAAACGTCT-TGGTTTA-ATTTCAAA----------------GCTTGACATATGGGTTCCACA
Mariner-11_HSal       TTATCGAATTCGACCGTGCATGAGCATTTGAAACAACT-TGGTTTC-GTTTCAAA----------------GCTCGATATTTGGGTTCCACA
Mariner-1_AFl         TTATCGAATTCGACTGTTCATGATCACATGAAACGACT-TGGTTTC-GTTTCGAA----------------GCTCGATATTTGGGTTCCACA
Mariner-36_HSal       GTATCACATACGAGCATTGAAAACCATTTAAAACAACT-TGGATAC-GTCAACAA----------------ACTCGATATTTGGGTTCCACA
Mariner-23_HSal       ATATCGATTGGAGCGTTTACCTTCATTTGAAACAACT-TGGTTAC-GTCAGTAA----------------GCTCGATGTTTGGGTCCCGCA
PBa(Mariner-23_HSal)  ATATCGATTGGAAGCGTTCACCTTCATTTGAAACAACT-TGGTTACAGTTAGTAA----------------GCTCGATATTTGGGTCCCGCA
Mariner-42_HSal       GTGTCACACAGAACAATTGAAAATCACATAAGACGTCT-TGGACTC-GTTAAGAA----------------GCTGGATATTTGGGTTCCGCA
Mariner-13_ACe        GTATCACACACAACAATTGAAAATCACTTGAAATGTTT-TGGAGTC-ATTAAAAA----------------GCTCGATATTTGGGTTCCTCA
AEc_AEVX01012963      GTATCACACACAACAATTGAAAATCACTTGAAATGTTT-TGGAGTC-ATTAAGAA----------------GCTCGATATTTGGGTTCCTCA
Mariner-2_HSal        GTATCACATACATGCATTGAAAAGCGTTTAAAACAACT-TGGCTAC-GTAAAAAA----------------ACTCGATTTATGGGTTCCTCA
AMe(FAMAR1)           GTATCACATACATGCATTGAAAACCACTTAAAACAACT-TGGCTAT-GTTCAAAA----------------ACTCGATACATGGGTTCCTCA
FAMAR1                GTATCACATACATGCATTGAAAATCACTTAAAACAACT-TGGCTAT-GTTCAAAA----------------ACTCGATACATGGGTTCCTCA
Mariner-1_BTe         GTATCGCATACATGCATTAAAAAA-AATTAAAACAGCT-TGGCTAT-GTCAAGAA----------------ACTCGATTTATGGGTCCCTCA
MARINER_CA            ATACCAAAATCAACCGTTCATTATCACATAAAAAGTCT-TGGACTG-GTGAAAAA----------------GCTTGATATTTGGGTACCACA
Mariner-1_ACe         GTGTCCGTTGGAAGTGTTCATGGCCATTTGAAATCGCT-AGGCTTC-GTAAAGAA----------------GCTCGATGTTTGGGTACCGCA
LHu_ADOQ01001582      GTGTCCATTGGAAGTGTTCATGGTCATTTGAAATCGCT-TGGTTTC-ATGAAGAA----------------GCTCGATGTTTGGGTATCGCA
LHu_ADOQ01008024      ATGTCCATTGGAAGTGTTCATGGTCATTTGAAATCGCT-TGGTTTC-ATGAAGAA----------------GCTCGATGTTTGGGTACCGCA
Mariner_Tbel          ATCCATCATTCAAGCGTTCACGACCACCTGAAGAAGCT-GGGATAC-GTAAGCAA----------------GCTCGATATTTGGGTTCCTCA
HSal(Mariner_Tbel)    ATCCATCATTCAAGCGTTCACGACCACCTGAAGAAGCT-GGGATAC-GTAAGCAA----------------GCTCGATATTTGGGTTCCTCA
PBa(Mariner_Tbel)     ATCCATCATTCAAGCGTTCACGACCACCTGAAGAAGCT-GGGATAC-GTAAGCAA----------------GCTCGATATTTGGGTTCCTCA
EEu(Mariner_Tbel)     ATCCATCATTCAAGCGTTCACGACCACCTGAAGAAGCT-GGGATAC-ATAAGCAA----------------GCTCGATATTTGGGTTCCTCA
CFl_(AEAB01001421)    ATCAGTCAAAAAAGTGT------CCACCTAAAGAAGCT-GGGATAC-GTAAGCAA----------------GATCGATGTTTGGGTTCCTCA
CFl_(AEAB01018477)    ATCAGTCAAAAAAGTGT------CCACCTAAAGAAGCT-GGGATAC-GTAAGCAA----------------GATCGATGTTTGGGTTCCTCA
SIn_(AEAQ01009575)    ATCCATCATGTAAGTGTTCACGGTCACTTGAAGAAGCT-GGGATAC-ATAAAGAA----------------GCTCGATATTTGGGTTTCTCA
SIn_(AEAQ01010279)    ATCCATCATTCAAGTCTTCACGACCGCCTGAAGAAGCT-GGGATAC-ATAAGCAA----------------GGTCATTATTTGGATTCCTCA
MRo_(AFJA01006902)    ATTCACCACTCAAGCGTTCATGACCACCTGAGGAAGCT-GGGTTAT-ATAAGCAG----------------GCTCGATGTGTGGGTTCCTCA
MRo_(AFJA01006736)    ATTCACCACTCAAGCGATCATGACCACCTAAAGAAGCT-GGGATAC-ATAAGCAGTGTGTACATAATGTACATAAGATGTGTGGGTTCCTTA
Mariner-22_HSal       ATATCGAAATCGAGCGTGGAAAATCATCTGCATCAACT-TGGTTAT-GTTAGCCG----------------TCTCGATGTTTGGGTTCCGCA
Mariner-35_HSal       GTATCTCAAAAGACCGTTGTGAACCACTTGCACACGCT-TGGCTAC-GTGTCTCG----------------GTACGATATTTGGGTACCTCA
Mariner1_BT           ATATCCAAATCAAGCGTTGAAAATCATTTGCACCAGCT-TGGTTAT-GTTAATCG----------------CTTTGATGTTTGGGTTCCACA
Mariner-5_ACe         ATGTCAAAATCCACCATCCACGAGCATTTTGTGAAGCT-TGGCTAC-ATAAACCG----------------TTTTGATGTATGGGTTCCCCA
Mariner-1_DF          GTGTCGCATACAACGGTTGAAAACCATTTGAAGAAACT-TGGCTAC-GTAAGCAA----------------GCTCGATGTTTGGGTTCCGCA
Mariner-6_CFl         ATTGGCAAATCAACTGTTTCCGAACATTTAAACAAGCT-CCAGATG-GTAAAAAA----------------GCTTGATGTTTGGGTGCCGCA
Mariner-16_HSal       ATTGACCAATCAACTGTTTCGAGACATTTACGGAAGCT-TGGGATG-GTAAACAA----------------GCTTGATGTTTGGGTGCCGCA
Mariner-47_HSal       ATATCCCACACAAGCGTAGAAAAGCATTTGCGCCAGCT-GGGATAC-GTTTCTCG----------------GCTTAATGTTTGGGTGCCGCA
Mariner-45_HSal       ATCCATCACCAAACAGTGTTAAACCATTTGGAGAAAGC-TGGCTAC-AAAAAGAA----------------GCTCGATGTTTGGGTGCCACA
AEc(Mariner-8_SIn)    ATCAACCATATGACAGTTTGGAATCATTTGAAAAAGGC-TGGCTAC-GCAAAGAA----------------ACTCGATGTTTGGGTGCCACA
Mariner-8_SIn         ATCAACCATGTGACAGTTTGGAATCATTTGAAAAAGGC-TGGCTAC-GCAAAGAA----------------ACTCGATGTTTGGGTGCCACA
Mariner-16_AEc        ATCGACCACAAAACAGTTTTAAATCATTTACACAAAGC-TGGATAC-AAAAAGAA----------------GCTCGATGTTTGGGTTCCTCA
Mariner-1_DEl         ATGAGTCATCAAACCGTTATAAACCATTTGAAGAAGCT-TGGAGTC-ACTAAGAA----------------GCTCGATGTATGGGTGCCACA
Mariner-46_HSal       ATTAGCCAGAAAACCGTTTGGAACCATTTGCATAAGGC-TGGTCTC-AAGAAGAA----------------GCTCGATGTATGGGTGCCACA
Mariner-2_DEl         ATTGCTCAAAAAACAGTTTGGAACCATTTAACAAAGGC-TGGATAC-ACAAAAAA----------------GCTCGATGTATGGGTGCCACA
Mariner-2_DEr         GTTAGTCACCAAACCATTTTAAACCATCTGCAGAAGGC-TGGATAC-AAAAAAAA----------------GCTTGATGTTTGGGTGCCGCA
Mariner-28_SIn        TTATCACATACAACCGTCATCGAGCATTTGCATAAGCTGTGGATAC-GTGAATCG----------------TCTTGATATCTGGGTGCCGCA
```

```
                    921
Mariner-24_SIn        TGTTCTTACGGAAAGAAATCTGTGCCGTCGCGTTGACGTCTGTGATTTGCTTCTCAAACGTCAAGAAAATGATCC-ATTTTTGAAGCGCATC
SMAR7                 TGTTCTTACAGAACGAAATTTGCTTCGTCGCATCAACGACTGTGATTTGCTTCTCAAACGTCAAGAAAATGATCC-ATTTTTGAAGTGAATC
Mariner-11_HSal       CGTTCTCAAAGAAAGAGATTTGATTCGTCGCATTACCATCTGCGATTTGCTGTTGAAACGTGAGGAAAACGATCC-ATTTCTGAAGCGACTC
Mariner-1_AFl         CGTTCTCAAAGAAAGAGATTTGCTCCGTCGCATTGATATCTGTGATTTGCTGCTCAAACGTGAGGAAAATGATCC-ATTTTTAAAGCGAATC
Mariner-36_HSal       CGAGCTCAAAGAAATTCATCTCACCAAACGCATCAATATCAGCGATTCTCTCCTGAAACGTGAGCAAAACGATCC-GTTTTTGAAACGAATG
Mariner-23_HSal       TGAACTGAAAGAAGTTCACCTCATAAAACGCATAGACATCTGCGATCAACTTCTGAAACGTGAACAATCCGACCC-ATTTCTGAAACGCATG
PBa(Mariner-23_HSal)  TGAACTGAAAGAAGTTCACCTCACTCAACGCATAGACATCTGCGATCAACTTATGAAACGTGAACAATCCGATCC-ATTTCTAAAACGTATG
Mariner-42_HSal       CGAATTGAAAGAAATTCACTTAACACAACGGATCAATATTTGCGATACGCATTTCAAACGTAATGCAATCGATCC-TTTTTTGAAGCGAATT
Mariner-13_ACe        TGAATTGAAAGAAATTCACTTAACACAACGAATCAACATTTGCGATTTACATCTCAAACGCAATAAAATCGACCC-TTTCTTGAAGCGAATT
AEc_AEVX01012963      TGAATTGAAAGAAATTCACTTAACACAACGAATCAACATTTGCGATTTGCATCTCAAACGCAATAAAATCGACCC-TTTCTTGAAGCGAATT
Mariner-2_HSal        TGAACTTAAAGAAATTCACTTAACACAACGCATTGCCATCTGCGATTTGCTTATGAAACGTAACGAAAATGATCC-ATTTTTGAAACGATTG
AMe(FAMAR1)           CGAACTGAAAGAAAAGCATTTAACGCAACGCATTAACAGCTGCGATTTGCTAAAGAAACGTAATGAAAATGATCC-ATTTTTAAAACGACTG
FAMAR1                CGAACTGAAAGAAACGCATTTAACGCAACGCATTAACATCTGCGATTTGCTAAAGAAACGTAATGAAAATGATCC-ATTTTTAAAACGACTG
Mariner-1_BTe         TCAGCTTAAGGAAATTCATTTGACGCAACGCATTAGCATCTGCGATTCGCTTCTGAAACGCAACGAAATTGATCC-ATTTCTGAAACGACTG
MARINER_CA            TGAATTGAAAGAAATTCATTTAACAAACCGAATCAACGCTTGTGATATGCATCTTAAACGCAATGAATTCGATCC-GTTTTTAAAGCGAATC
Mariner-1_ACe         TGAGTTGAAAGAAATTCATCTGACGAATCGCATGAGCGTCTGCGATCAACTCATCAAACGCGAAGAAAACGATCC-GTTCTTGAAGCGTATG
LHu_ADOQ01001582      TGAGTTGAAAGAAATTCATCTGACGAAGCGTATGAACGTCTGCGATCAACTCACCAAACGCGAAAAAAACGATCC-GTTCTTGAAGCGTATG
LHu_ADOQ01008024      TGAGTTGAAAGAAATTCATCTGACGAAGCGTATGAACGTCTGCGATCAACTTACCAAACGCGAAAAAAACGATCC-GTTCTTGAAGCGTATG
Mariner_Tbel          TGAACTCAAAGAAGTTCATTTGACGGCGCATATAAACATCTGCGATATGCTCATTAAACGTGAGGAAAACGATCC-TTTTTTGAAGCGACTG
HSal(Mariner_Tbel)    TGAACTCACAGAAGTTCATTTGACGGCGCGTATAAACATCTGCGATATGCTCATTAAACGTGAGGAAAACGATCC-TTTTTTGAAGCGAATG
PBa(Mariner_Tbel)     TGAACTCAAAGAAGTTCATTTGACGGCGCGTATAAACATCTGCGATATGCTCATTAAACGTGAGGAAAACGATCC-TTTTTTGAAGCGACTG
EEu(Mariner_Tbel)     TGAACTCAAAGAAGTTCATTTGACGGCGCATATAAACATCTGCGATATGCTCATTAAACGTGAGGAAAATGATCC-TTTTTTGAAGTGACTG
CFl_(AEAB01001421)    TGAACTCAAAGAAGTTCATTTGACGGCGCGTATAAACATCTGCGATATGCTCATAAAACGTGAAGAAAACGATCC-TTTTTTAAAACGAATG
CFl_(AEAB01018477)    TGAACTCAAAGAAGTTCATTTGACGGCGCGTATAAACATCTGCGATATGCTCATAAAACGTGAAGAAAACGATCC-TTTTTTAAAACGAATG
SIn_(AEAQ01009575)    TGAACTCAAAAAAGTTTATTTGATGGTGCGTATAAACATCTGCAATAT-----------GTGAAGAAAATGATCCTTTTTTTGAAGCGACTG
SIn_(AEAQ01010279)    TGAACTCAAAGAAGTTTATTTGACGGCGCGTACAAATATTTGCAATATGCTCATTAAGCGTAAGGAAAACAAATC-TTTTTTGAAGCGACCG
MRo_(AFJA01006902)    TGAGCTTAAAGAGGTTCATTTGACGGCGCGTGTAAGCATCTGCGACACGCTCATCAAACGGCAAAAAAACGACCCTTTTTTTGAGACGGCTA
MRo_(AFJA01006736)    TGAGCTCAAAGAGGTTCATTTGACTGCGCGTATAAGCATCTGCGATACGCTTATCAAACGGCAGAAAAACGACCC-TTTTTTGAAGCGGCTA
Mariner-22_HSal       CGAACTGAGCGAAGCTCATTTAATCCAACGCATTTCCATCAGCGATTCACTCGGAAAACGTGAGAAAAGCGATCC-ATTTCTGAAGCGTATG
Mariner-35_HSal       CAATTTGAGCGAAAAAAATTTAATGGACCGCATCTCCATCTGCGATTTATTGCTCAAACGCAATGAAAACGTTCC-ATTTTTAAAACGGATG
Mariner1_BT           TAAGTTAAGCGAAAAAAACCTTCTTGACCGTATTTCCGCGTGCGATTCTCTACTGAAACGTAACGAAAACGTTCC-GTTTTTAAAACAAATT
Mariner-5_ACe         CGATTTAACGGAGAAAAATCTGATGGATCGCATTTCCATTTGCGACTCGCTCTATAAACGCAACGAGGAGACACC-ATTTTTGAAGCAAGTA
Mariner-1_DF          TAACTTAAAGGAAATTCACCTAACTAAGCGTATCAACATCTGCTATTCTCTGCTGCAACGGATTAAAAACGATCC-TTTTTTGAAGCGGATC
Mariner-6_CFl         CGACTTAACTGAGAAAAATCGTATTGACCGCATTTCCATCAGCGATTTGCTATACAAACGCAATGAATACGATTC-ATTTTTAAAACGCATC
Mariner-16_HSal       CGAACTGAGCGAAAAGAATTTAATGGACCGAATTTCAGCTTGCGATTTGCTGCTCAAACGCCATCAAAACGAACC-ATTTTTAAAGCGGATG
Mariner-47_HSal       TCAATTAACCGAGGCTAACTTGACCACACGCATATCCATCTGCGATTCGCTACGGAAACGCCAAGAAAACGACCC-ATTTTTTAAACGAATG
Mariner-45_HSal       TGATCTGACTCAAAAAAATTTACTCGATCGAATTTCTATCTGCGAATCTCTACTGAAACGTAACGAAATCGAGCC-ATTTCTGAAGCGGTTG
AEc(Mariner-8_SIn)    TGAATTAACGCAAAGAAATTTGATTGATCGAATTTCCATCAGCGAAACACTACTAAAACGCAACGAAATCGACCC-ATTTCTGAAGCAAATC
Mariner-8_SIn         TGAATTAACGCAAAGAAATTTGATTGACCGAATTTCCATCAGCGAAACACTACTAAAACGCAACGAAATCGACCC-ATTTCTGAAGCAAATC
Mariner-16_AEc        CGAGTTGAGTGTTAAGAACATGATGGACCGAATTAACATTTGCGATACGCTACTGAAACGGAATGAAATTGAGCC-ATTTCTGAAGAGAATG
Mariner-1_DEl         CGAATTGACGCAAAAAAACATCTTTGACCGTATCGACGCATGCGAATCGCTTCTAAATCGCAACAAAATCGACCC-GTTTTTGAAGCGTATG
Mariner-46_HSal       CGAATTGACGCAAAAGAACCTTTTGGACCGAATTCATGCCTGCGATTCTTTGCTGAAGCGTAACGAAATCGACCC-ATTTTTGAAGCGGATG
Mariner-2_DEl         TGAGCTA--TAAAAAAAACCTTTTGGACCGAATTTCGATCTGCGAATCGCTGCTGAATCGCAACAAAACCGACCC-GTTTTTGAAGCGGATG
Mariner-2_DEr         TGATTTGACGCAAAAAAACCTTCTGGACCGAATCAACGCCTGCGATATGCTGCTGAAACGGAACGAACTCGACCC-ATTCTTGAAGCGGATG
Mariner-28_SIn        TGATTTGTCTGACAAAAATTTAATTGATCGCATTTCCATCTGTGATTTGTTACTTAAACGCAACGAAAA--AATC-CATTTTTAAACGAATC

                   1013
Mariner-24_SIn        ATCACTGGGGACGAGAAATGG-----GTTGTC----------------------------------------------------TACAACAAT
SMAR7                 ATTACTGGCGACGAGAAATGAGGGTTGTTRTT----------------------------------------------------TACAAAAAT
Mariner-11_HSal       ATAACTGGAGATGAAAAATGG-----ATTGTG----------------------------------------------------TACAACAAT
Mariner-1_AFl         GTAACTGGAGATGAAAAGTGG-----ATCGTT----------------------------------------------------TACGACAAT
Mariner-36_HSal       TTAACTGGCGATGAAAAATGG-----ATTGTT----------------------------------------------------TACGACAAT
Mariner-23_HSal       ATTACTGGCGATGAAAAATGG-----ATTGTC----------------------------------------------------TACAACAAT
PBa(Mariner-23_HSal)  ATTACTGGCGATGAAAAATGG-----ATCGTG----------------------------------------------------TACAATAAT
Mariner-42_HSal       ATCACTGGTGATGAAAAATGG-----ATCGTC----------------------------------------------------TACAACAAC
Mariner-13_ACe        ATTACGGGTGATGAAAAATGG-----ATCGTC----------------------------------------------------TATAATAAC
AEc_AEVX01012963      ATTACGGGTGATGAAAAATGG-----ATCGTC----------------------------------------------------TATAATAAC
Mariner-2_HSal        ATAACTGGCGATGAAAAATGG-----GTTGTT----------------------------------------------------TACAACAAT
AMe(FAMAR1)           ATAACTGGCGATGAAAAATGG-----GTTGTT----------------------------------------------------TACAACAAT
FAMAR1                ATAACTGGCGATGAAAAATGG-----GTTGTT----------------------------------------------------TACAACAAT
Mariner-1_BTe         ATTACTGGCGACCAAAAGTGG-----ATAGTT----------------------------------------------------TATAACAAC
MARINER_CA            ATAACTGGAGATGAAAAATGG-----ATTGTT----------------------------------------------------TACAATAAC
Mariner-1_ACe         ATCACGGGCGACGAAAAATGG-----ATTGTT----------------------------------------------------TACAACAAT
LHu_ADOQ01001582      ATCACGGACGACGAAAAATGG-----ATTGTC----------------------------------------------------TACAACAAT
LHu_ADOQ01008024      ATCACGGGCGACGAAAAATGG-----ATTGTC----------------------------------------------------TACAACAAT
Mariner_Tbel          ATAACTGGTGATGAGAAGTGG-----ATTGTT----------------------------------------------------TACAACAAT
HSal(Mariner_Tbel)    ATAACTGGTGATGAGAAGTGG-----ATTGTT----------------------------------------------------TACAACAAT
PBa(Mariner_Tbel)     ATAACTGGTGATGAGAAGTGG-----ATTGTT----------------------------------------------------TACAACAAT
EEu(Mariner_Tbel)     ATAACTGGTGATGAGAAGTGG-----ATTGTT----------------------------------------------------TACAACAAT
CFl_(AEAB01001421)    ATAACTGGCGATGAGAAGTGG-----ATTGTT----------------------------------------------------TACAACAAT
CFl_(AEAB01018477)    ATAACTGGCGATGAGAAGTGG-----ATTGTT----------------------------------------------------TACAACAAT
SIn_(AEAQ01009575)    ATAACTGATGATAAGAAATGG-----ATTGTT----------------------------------------------------TACAACAAT
SIn_(AEAQ01010279)    -TAACTGGTGTTGAGAAGTAA-----ATTGTTTGAGGCAAATCTTCACGTCGGAATCAAGACCGTTTGCGCTCTACATTGTTGTACAACAAT
MRo_(AFJA01006902)    ATAACTGGCGATGAAAAATGG-----ATAGTT----------------------------------------------------TACAACAAT
MRo_(AFJA01006736)    ATAACTGGTAATGAAAAATGG-----ATTGTT----------------------------------------------------TACAACAAT
Mariner-22_HSal       GTAACTGGCGACGAAAAATGG-----ATCGTC----------------------------------------------------TACAATAAC
Mariner-35_HSal       ATAACGGGCGACGAAAAGTGG-----ATTGTG----------------------------------------------------TATAACAAT
Mariner1_BT           GTGACGGGCGATGAAAAGTGG-----ATACTG----------------------------------------------------TACAATAAT
Mariner-5_ACe         GTGACGGGGGATGAAAAATGG-----ATCATT----------------------------------------------------TACAATAAT
Mariner-1_DF          ATTACTGGCGACGAAAAGTGG-----GTTGTT----------------------------------------------------TACAATAAT
Mariner-6_CFl         ATAACAGGCGACGAAAAGTGG-----ATAATT----------------------------------------------------TATAATAAT
Mariner-16_HSal       ATCACTGGCGATGAAAAGTGG-----ATTGTC----------------------------------------------------TACAACAAT
Mariner-47_HSal       GTGACGGGTGACGAAAAATGG-----GTCGTC----------------------------------------------------TACGATAAT
Mariner-45_HSal       ATCACGAGTGATGAAAAGTGG-----ATAACC----------------------------------------------------TACGACAAC
AEc(Mariner-8_SIn)    ATCACAGGCGATGAAAAATGG-----GTGAAA----------------------------------------------------TACAAGAAT
Mariner-8_SIn         ATCACGGGCGATGAAAAATGG-----GTAAAA----------------------------------------------------TACAAGAAT
Mariner-16_AEc        ATAACTGGTGATGAAAAATGG-----ATCACG----------------------------------------------------TACGACAAT
Mariner-1_DEl         GTGACTGGCGATGAAAAGTGG-----GTCACT----------------------------------------------------TACGATAAC
Mariner-46_HSal       GTGACTGGCGATGAAAAATGG-----GTCACA----------------------------------------------------TACGAGAAC
Mariner-2_DEl         GTGACTGGTGATGAAAAGTGG-----GTCACT----------------------------------------------------TACGACAAC
Mariner-2_DEr         GTGACTGGCGACGAAAAATGG-----ATCACA----------------------------------------------------TACGACAAT
Mariner-28_SIn        GTGACGGGCGATGAAAAATGG-------------------------------------------------------------------------
```

```
                         1105
Mariner-24_SIn          GTTAAACGCAAGAGATCATGGAGTAAAAAAGATGAACCGGCTCAAAGCACTTCGAAAGCCGATATTCATCAAAAAAAGGTGAT------GCT
SMAR7                   GTTAAGCGCAAGAGATCATGGAGTAAAAAAGATGAACCAGCTCAATCCACTTCGAAGGCCGATATTCACCAAAAAAAGGTTAT------GCT
Mariner-11_HSal         ATTAAACGCAAAAGATCATGGAGCAGACGTGATGAACCAGCTCAAAGCACATCGAAAGCTGATATCCATCAAAAGAAGGTGAT------GCT
Mariner-1_AFl           GTTAAACGAGAGTA-------------GAGATGAGCCGGCGCAAAGCACTTCGAAAGCCAACATTCACCAAAAAAAAGTGAT------GCT
Mariner-36_HSal         GTGGAACGAAAAAGATCGTGGAGCAAACGTGATGAGCCGCCACAAAGCACTTCTAAAGCAGACATTCACCAAAGGAAGATTTT------GCT
Mariner-23_HSal         GTGAAGCGAAAGCGATCGTGGAGCAAGCGCGATGAGCCGGCCCAAAGCACATCAAAAGCAGACATCCACCAGAGAAAGGTGAT------GCT
PBa(Mariner-23_HSal)    GTGGAGCGAAAGCGATCGTGGAGCAAACCCGATGAGCCGGCCCAAAGCACATCAAAAGCAAACATCCACCAGAGGAAAGTGAT------GCT
Mariner-42_HSal         ATTAATCGAAAAGATCATGGTCCAAGCATGATGAACCGGCACAAACCGTATCGAAAGCTGAATTGCATCAAAAAAGATCAT------GCT
Mariner-13_ACe          ACCAATCGAAAACGATCATGGTCTAAGCCTAATGAACCAGCACAAACCTCATCGAAAGCCGAACTGCATCAAAAAAAGATAAT------GCT
AEc_AEVX01012963        ACCAATCGAAAACGATCATGGTCTAAGCCTAATGAACAAGCACAAACCTCATCGAAAGCTGAACTGCATCAAAAAAAGATAAT------GCT
Mariner-2_HSal          GTCAATCGAAAAAGATCGTGGAGCAAGCAAGATGAACCAGCGCAAACAACATCCAAAGCTGATATTCATCAAAAGAAAGTTAT------GTT
AMe(FAMAR1)             ATCAAGCGGAAAAGATCGTGGAGCAGGCCACGTGAACCAGCTCAAACAACATCAAAAGCTGGTATTCATCAAAAGAAGGTTTT------GTT
FAMAR1                  ATCAAGCGGAAAAGATCGTGGAGCAGGCCAGGTGAACCAGCTCAAACAACATCAAAAGCTGGTATTCATCAAAAGAAGGTTTT------GTT
Mariner-1_BTe           GTTAATCGGAAAAGATCGTGGGTGATGCAAGATGAACCAGCCCAGACGACACCAAAAGCTGAGATTCACCA-AAAAAGATTAT------GCT
MARINER_CA              GTTAATCGAAAACGATCATGGTCCAAGCATGGTGAACCAGCTCAAACCACTTCAAAGGCTGATATCCACCAAAAGAAGGTTAT------GCT
Mariner-1_ACe           GTCAGCCGAAAAAGAAGTTGGAGTAGGCGAGGTGAAGCACCGGAAAGGCAAGCAAAGGCGGAGATCCACCAAAAGAAGGTAAT------GCT
LHu_ADOQ01001582        GTGAACCGAAAAAGGAGTTGGAGTAGGCGAGATGAAGCACCGGAAAGGCAAGCAAAGGCGGACATCCACCAAAAGAAGGTGAT------GCT
LHu_ADOQ01008024        GTGAACCGAAAAAGGAGTTGGAGTAGGCGAGATGAAGCACCGG----------AAAGGCGGACATCCACCAAAAGAAGGTGAT------GCT
Mariner_Tbel            GTGGTGCGCAAACGGTCTTGGTCCCGACGTGATGATTCGCCTCAAACGACATCGAAAGCAGACATTCATCAGAGGAAGGTTAT------GCT
HSal(Mariner_Tbel)      GTGGAGCGCAAACGGTCTTGGTCCCGACGTGATGATTCGCCTCAAACGACATCGAAAGCAGACATTCATCAGAGGAAGGTTAT------GCT
PBa(Mariner_Tbel)       GTGGTGCGCAAACGGTCTTGGTCCCGACGTGATGATTCGCCTCAAACGACATCAAAAGCAAACATTCATCAGAGGAAGGTTAT------GCT
EEu(Mariner_Tbel)       GTGGTGTGCAAACAGTCTTGGTCCCGATGTGATGATTCACCTCAAATGACATCGAAAGCAGACATTCATCAGAGGAAGGTTAT------GCT
CFl_(AEAB01001421)      GTGGAACGCAAACGGTCTTGGTCCCGACGTGATAATTCGCTTCAAACAACTTCAAAAGCAGAACTTCATCAGAGGAAGATTAT------GCT
CFl_(AEAB01018477)      GTGGAACGCAAACGGTCTTGGTCCCGACGTGATAATTCGCTTCAAACAACTTCAAAAGCAGAACTTCATCAGAGGAAGATTAT------GCT
SIn_(AEAQ01009575)      TTGGAGCGCA----GTCTTAGTCCCGGCGTAATGATTTGTCTCAAACGACATCAAAAGCAAACAT---TCAGAGGAAAGTTAT------GCT
SIn_(AEAQ01010279)      GTAGAACGCAAATGGTTTTGGTCCCGATGTGAAGATTTGCCTCAAACGACATCAAAAGCAAACATTCATCAGAGAAAAATTATGTTGTGGCT
MRo_(AFJA01006902)      ATAATGCGCAAGCGGTCTTGGTCTCAACGTGAAGTACCGCTTCAAACGAGATCGAAACCAGAGATCCACCAGAGGTAGATTAT------GCT
MRo_(AFJA01006736)      ATAGTGCGTAAACGGTCTTGGTCTCAACGTGATGCACTGCCTCAAACGAGATCGAAACCAGAGATCCACCACAGGAAGGTAAT------GCT
Mariner-22_HSal         ATCAAGCGCAAAAGATCGTGGGGGCAGCGCAGCGAACCGCCACAAACCACTTCGAAGGCAGGGCTTCACCCGAGCAAGATCAT------GCT
Mariner-35_HSal         GTAGAGCGCAAAAGATCGTGGGAAACGAGATGAACCGCCGCAAACCACACCGAAGCAAACATCCATACGAAAAAAGTCAT------GCT
Mariner1_BT             GTGGAACGGAAGAGATCGTGGGGCAAGCGAAATGAACCACCACCAACCAACACCAAAGGCCGGTCTTCATCCAAAGAAGGTGAT------GTT
Mariner-5_ACe           GTTGAGCGAAAAAGATCTTGGGGAAAGCGGAATGAACCACCTTTAGCCACCCCAAAAGCCGGTCTTCATCCAAAGAAGGTCAT------GCT
Mariner-1_DF            GTCCAACGAAAAAGATCATGGAGCAAGAAAGATGAGCCAGCCCAAGCCACATCAAAGGCCGATATCCATCAGAGGAAAGTTAT------GCT
Mariner-6_CFl           GTCGAGCGTAAAAGGTCGTGGAGCAAGCGAGGTGAACCGCCATTAACCACTTCGAAGGCAGGCTTACATCCGAAAAAGGTAAT------GCT
Mariner-16_HSal         GTCAGTCGAAAAAGATCATGGTCGAAGCGCGGGGAGGCTGCTCAGACGGTCGCAAAGGCTGGATTACACCCGAAGAAGGTCAT------GCT
Mariner-47_HSal         GTAACGCGTAAAAGATCATGGGGGCACAGCAGCGAGCCACCGCAAACAACCTCGAAGGCGGGATTGCATCCGAAGAAAATAAT------GCT
Mariner-45_HSal         AATGTGCGAAAAAGATCGTGGTCGAAGCGAGGAGAAGCTCCACAGTCTGTGGCAAAGCCCACACTGACGCCCAGGAAGGTTAT------GCT
AEc(Mariner-8_SIn)      ATTGTTCGTAAAAGATCATGGAATAAGCGCGGCGAACCACCACAAACGACTTCAAAGCCTGGATTGATGGCAAATAAGGTGAT------GCT
Mariner-8_SIn           ATCGTTCGTAAAAGATCATGGGGTAAGCGCGGCGAACCACCACAAACGACTTCAAAGCCTGATTTGACGACAAATAAGGTGAT------GCT
Mariner-16_AEc          CGAACTCGCAAAAGATCGTGGATAAAGGAAGGAGAGAAGGCACAAGCAATCGCAAAACCTGGATTGACGACGAAGAAGGTGAT------GTT
Mariner-1_DEl           GTAAGGCGCAAACGGTCGTGGTCGAAAAGCGGTGAAGCGGCCCAGACGGTGGCCAAGCCTGGATTGACGGCCAGGAAGGTTCT------TCT
Mariner-46_HSal         AACAGGCGAAAAAGATCGTGGTCCAAGCGCGGTGAGCCGGTCCAAACGATCGCCAAGCCCGGATTAACGGCCAAGAAGGTTTT------ACT
Mariner-2_DEl           GTGAGGCGAAAACGGTCGTGGTCGAAGAAGGATGAGCCGGCCCAGGCGGTGGCTAAGCCAGGATTGACGGCCAGAAAGGTTTT------GCT
Mariner-2_DEr           ATCAAGCGAAAACGGTCGTGGTCGAAGGCCGGTGAATCGTCCCAAACAGTGGCCAAGCCGGGATTGACGGCCAGGAAGGTTTT------GCT
Mariner-28_SIn          -------------GTCTTGAGGAAAACATTCAGAAAAGCCGTTGACCACTCCAAAGCCTGGGCTTCATCCGGAGAAGATCAT------GCT

                         1197
Mariner-24_SIn          ATCTGTTTGGTGGGATTTCAAAGAAATCTTTTTTTTTTTGAGCTTCTACCGGACAACACAACGATCAATTCTGAAGTGTACTGTCATCAGCT
SMAR7                   ATCTGTTTGGTGGGATTTCAAGGGGATC---GTTTTTTTGAGCTTCTACCGGACAATACCACGATTAATTCTGAAGTGTACTGTGATCAACT
Mariner-11_HSal         CTCTGTTTGGTGGGATTGGAAAGGAATG--GTTTTTTTTGAGCTGCTACCAAACAACCAAACGATTGATTCGAATGTGTATTGTCATCAATT
Mariner-1_AFl           CTCTGTTTGGTGGGATTTCAAGGGAGTT--GTTTTTTTTGAGCTTCTACCTGACAATTGCACGATTAATTCGGAAGTGTACTGCAATCAATT
Mariner-36_HSal         ATCTGTTTGGTGGGATTACAAGGGTATT--GTGTTTTTTGAACTGCTTAAACGTAATCAAACGATTGATTCGGAATTGTACTGTCGTCAATT
Mariner-23_HSal         CTCAATCTGGTGGGATTGGAAGGGTGTG--GTGTTTTTCGAGCTCTTACCAAGGAATGTTACGATCAATTCGGACGTGTACTGTCAACAGCT
PBa(Mariner-23_HSal)    CTCAATCTGATGGGATTGGAAGGGTGTG--GTGTTTTTCGAGCTCTTACCAAGGAATGTTTCGATCAATTCGGACGTGTACTGTCAACAGCT
Mariner-42_HSal         GTCAATTTGGTGGGATTACAAAGGTGTT--GTGTATTTTGAGATGCTTCCAAGCAACCAAACGATCAACTCAGATGTTTACTGCCAACAACT
Mariner-13_ACe          GTCAATTTGGTGGGATTTCAAAGGTGTT--GTGTATTTTGAGCTGCTTCCAAGGAATCAAACGATTAATTCAGATGTCTACTGCCAACAACT
AEc_AEVX01012963        GTCAATTTGGTGGGATTTCAAAGGTGTT--GTGTATTTTGAGCTGCTTCCAAGGAATCAAACGATTAATTCAGATGTCTACTGCCAACAACT
Mariner-2_HSal          ATCAGTTTGGTGGGATTACAAAGGAATT--GTCTACTTTGAAATTTTACCACGCAACCAAACGATCAATTCAGATGTATACATTCAACAACT
AMe(FAMAR1)             ATCAGTTTGGTGGGATTACAAAGGAATT--GTCTATTTTGAACTCTTACCACCCAACCGAACGATCAATTCTGTTGTCTACATTGAACAACT
FAMAR1                  ATCAGTTTGGTGGGATTACAAAGGAATT--GTCTACTTTGAACTCTTACCACCCAACCGAACGATCAATTCTGTTGTCTACATTGAACAACT
Mariner-1_BTe           GTCAGTTTGGTGGGATTATAAAGGAATT--CTGTACTTTGAACTTTTACCAAGAAACCAAACGATTAATTCAAACGTGTACGTTCAACAACT
MARINER_CA              GTCTGTTTGGTGGGATTGGAAGGGTGTG--GTATATTTTGAGCTGCTTCCAAGGAACCAAACGATTAATTCGGATGTTTACTGTCAACAACT
Mariner-1_ACe           GTCAATGTGGTGGGATTGGAAGGGACCA--GTCTTCTATGAGCTACTTCCAAAGAACAAAACGATTAATTCCGATGTCTACTGTGAGCAGCT
LHu_ADOQ01001582        GTCAATCTGGTGGGATTGGAAGGGACCA--GTCTTCTATGAGCTGCTTCCAAAGAATAAAACGATTAATTCCGATGTCTCCTGTGAGCAGCT
LHu_ADOQ01008024        GTCAATCTGGTGGGATTGGAAGGGACCA--GTCTTCTATGAGCTGCTTCCAAAGAATAAAACGATTAATTCCGATGTCTACTGTGAGCAGCT
Mariner_Tbel            CTCAGTGTGGTGGGATTTTAAAGGTGTC--GTGTTTTTCGAGCTCCTACCAAGGAACCAAACAATCAATTCGAATGTGTACTGTCGGCAATT
HSal(Mariner_Tbel)      CTCAGTGTGGTGGGATTTTAAAGGTGTC--GTGTTTTTCGAGCTCCTACCAAGGAACCAAACAATCAATTCGAATGTGTACTGTCGGCAATT
PBa(Mariner_Tbel)       CTCAGTGTGGTGGGATTTTAAAGGTGTC--GTGTTTTTCGAGCTCCTACCAAGGAACCAAACAATCAATTCGAATGTGTACTGTCGGCAATT
EEu(Mariner_Tbel)       CTCAGTGTGGTGGGATTTTAAAG-------GCATTTTTTGAGCTCCTACCAAGGAACCAAACAATCAATTCAAATGTGTACTGTCGGCAATT
CFl_(AEAB01001421)      CTCAGTGTGGTGGGATTGGAAGGGTGTG--GTGTTTTTTGAGCCCCTACCAAGGAACCGAACAATCAATTCGGATGTGTACTGTCGGCAATT
CFl_(AEAB01018477)      CTCAGTGTGGTGGGATTGGAAGGGTGTG--GTGTTTTTTGAGCCCCTACCAAGGAACCGAACAATCAATTCGGATGTGTACTGTCGGCAATT
SIn_(AEAQ01009575)      CTTAATGTGATGGAATTTTAAAGGTGTC--GTGTTTTTCGAGCTCCCACCAAGAAACCAAATAATCAATTCGCGTATGTACTGTCGGCAATT
SIn_(AEAQ01010279)      CTCAGTGTGGTGAGATGTTAAAGGTATC--GTGTTTCTCGAGCTCCTACCAAGGAACCAAACAATCAATTGAAACGTGTACTGTTGGCAATT
MRo_(AFJA01006902)      CTCTGTGTGGTGGGATTTCAAAGGCACA--GTGTTTTCCGAACTCTTATCAAGAAATCAAACGATCAATTCTGACGTCTACTGCCGGCAATT
MRo_(AFJA01006736)      CTCTGTGTGGTGGGATTTGAAAGGCATA--GTGTTTTTCGAATTCCTATCAAGAAATCAAACGATCAATTCTGATATCTACTGCCGGTAATT
Mariner-22_HSal         CTCAATTTGGTGGGATTGGAAGGGTGTT--GTGTTCTACGAGCTACTTCCGAAGAACAGAACGATCAATTCAGATGTGTACTGTAGTCAGCT
Mariner-35_HSal         GTGTATTTGGTGGGATTGGAAAGGAATC--GTGTACTACGAGCTGCTTCCGCAAAACCAAACAATTGATTCCAACAAGTATTGTTCCCAATT
Mariner1_BT             GTGTATATGGTGGGATTGGAAGGGAGTC--CTCTATTATGAGCTCCTTCCGGAAAACCAAACGATTAATTCCAACAAGTACTGCTCCCAGTT
Mariner-5_ACe           CTGTGTCTGGTGGGATTGGAAAGGGATC--CTGTATTATGAGCTTTTACCAAACAACGAGACGATAAATTCAGAGAAGTATTGTTCCCAATT
Mariner-1_DF            GTCTGTTTGGTGGAATTGGAAGGGTGTA--GTGCACTTTGAGCTGCTGGGACGGAATGAAACCATCAATTCAGATGTGTACTGTCGCCAGCT
Mariner-6_CFl           CTGCATTTGGTGGAATTGGAAGGGGATA--GTATATTATGAGCTACTACCGGACAACGAAACCATCGATTCGGACAAGTACTGTTCACAGTT
Mariner-16_HSal         CTGTATCTGGTGGGATTGGAAAGGAATC--GTCTATTATGAGCTGCTGCCACCCAACAAAACGATTGATTCGACCAAGTACTGCTCACAACT
Mariner-47_HSal         CTCCGTATGGTGGGATTTCAAGGGTGTC--ATCTATTACGAACTGTTACCGTCAGGCAAAACGATTGATTCAACTGTATACTGTTCGCAATT
Mariner-45_HSal         GTGTGTTTGGTGGGATTGGAAGGGCATC--GTGCATCACGAGCTGCTGCCGCCAGGCAGAACGATAGACTCGGACCTGTACTGTCGACAATT
AEc(Mariner-8_SIn)      GTGTGTATGGTGGGATTGGAGGGAGTC--GTCCATCATGAGGTGTTACCACATGGCCTAACGATTAATTCAGAGCTCTACTGTTCACAACT
Mariner-8_SIn           GTGTGTATGGTGGGATTGGAAGGGAGTC--GTCCATTATGAGGTGTTACCACATGGCCTAACGATTAATTCAGAGCTCTACTGTTCACAACT
Mariner-16_AEc          GTGTGTGTGGTGGGATTGGAAGGGAATC--GTTCACTATGAGCTGTTATCGCCCGATCAAACAATTAATTCCGAACTCTACTGTGAACAACT
Mariner-1_DEl           GTGTGTTTGGTGGGATTGGCAGGGAATA--ATCCACTATGAGCTGCTCCCCTATGGCCAAACGCTCAATTCGGACCTGTACTGCCAACAACT
Mariner-46_HSal         GTGTGTTTGGTGGGATTGGAAGGGAGTC--ATCCACTATGAGCTGCTCCCATCGGGCCAATCACTCAATTCGGACCTACACTGTCAACAACT
Mariner-2_DEl           GTGTGTTTGGTGGGATTGGCAAGGAATT--ATCTACTATGAGCTGCTCCCCTATGGCCAAACACTTAATTCGGTTTTGTACTGTCAACAATT
Mariner-2_DEr           GTGTGTTTGGTGGGATTGGAAGGGAATC--ATCCACTATGAGCTGCTCCCATATGGCCAGACGCTTAATTCTACCATCTACTGCGAACAACT
Mariner-28_SIn          GTGTATTTGATGGGACTGGAA-GGTGTC--GTGTATTATGAGCTCCTTCCAAAGAACGTACGGCTTAATTCGGACAAGTACTGTGCCCAATT
```

```
                          1289
Mariner-24_SIn         GGACAAATTGAATGATTCACTTAAACAGAAAAG------GCCAGAATTGATCAATAGAAAAGGTGTAGTGTTCCACCAAGATAATGCGAGAC
SMAR7                  GGACAAATTGAATGATTCGCTCAAACAGAAAAG------GCCAGAATTGATCAATAGAAAAGGTATAGTGTTCCACCACGATAATGCGAGAC
Mariner-11_HSal        GGACAAATTGAATGATTCCATCAAGCAGAAGAG------ACCAGAATTGGCGAACAGAAAAGGTGTTGTGTTTCATCATGACAACGCCAGAC
Mariner-1_AFl          GGACAAATTAAACGATTCCATCAAACAAAAGAG------ATCTGAATTAATTAACAGGAAAGATGTAGTGTTTCATCACGATAACGCTAGAC
Mariner-36_HSal        GGACAAATTACACGAAGCAATCAAGCAGAAGCG------CCCAGAACTGGTGAATCGTAAAGATGTTGTCTTTCACCATGACAACGCTAGAC
Mariner-23_HSal        CGACAAATTGAACGAAGCAATAGCAGAAAAGCG------GCCAGAATTGATAAATCGCAAAGGAGTGGTCTTCCATCATGACAACGCTCGAC
PBa(Mariner-23_HSal)   AGACAAACTGAACGAAGCAATAGCAGAAAAGCG------GCCAGAATTGATAAATCGCAAAGGAGTAGTATTCCAACATGACAACGCTCGAC
Mariner-42_HSal        TATGAAATTGGAGGAAGCAATCAAAGAGAAAAG------GCCAGAATTGGCAAATCGTAAAGGAATCGTGTTCCACCACGACAATGCGAGGC
Mariner-13_ACe         AATGAAACTGGAGGAAGCAATCAAAGAAAAACG------GCCAGAGTTAGCGAATCGCAAAGGAATCGTCTTTCATCATGACAATGCAAGAC
AEc_AEVX01012963       AATGAAACTGGAGGAAGCAATTAAAGAAAAACG------GCCAGAGTTAGCGAATTGCAAAGGAATCGTCTTTCATCATGACGATGCAAGAC
Mariner-2_HSal         AACCAAACTGAACAATGCAATTCAAGAAAAGCG------ACCAGAATTGGCAAATCGAAAAGGTATTGTGTTCCACCATGACAATGCAAGGC
AMe(FAMAR1)            AACGAAATTAAACAATGCAGTTGAAGAAAAGCG------GCCCGAATTGACAAATCGAAAAGGTGTTGTATTCCATCATGACAATGCAAGGC
FAMAR1                 AACGAAATTAAACAATGCAGTTGAAGAAAAGCG------GGCCGAATTGACAAATCGAAAAGGTGTTGTATTCCATCATGACAATGCAAGGC
Mariner-1_BTe          CGCCAAACTGAGCGATGCAGTTCAAGAAAAGCG------GCCAGAATTGGCAAATCGTAAGGGTGTTGTTTTCCAGCATGATAATGCAAAGC
MARINER_CA             GGACAAATTGAATGCAGCCATCAACGAGAAACG------GCCAGAATTGATCAATCGTAAAGGTGTCATATTCCATCAGGACAACGCCAGAC
Mariner-1_ACe          GCAGAAATTAAGTGATGCCATCGCACAGAAACG------TCCCGAGCTAATCAATCGTAAGGGTGTGGTGTTTCACCACGACAATGCGAGAC
LHu_ADOQ01001582       ACAGAAATTAAGTGATGCCATCGCACAGAAACG------TCCCGAGCTAATCAATCGTAAGGGTGTGGTGTTTCACCACGACAATGCGAGAC
LHu_ADOQ01008024       ACAGAAATTAAGTGATGCCATCGCACAGAAACG------TCCCGAGCTGATCAATCGTAAGGGTGTGGTGTTTCACCACGACAATGTGAGAC
Mariner_Tbel           GGACAGTTTGAACGAATCCATCATCCAGAAACG------TCCAGAGCTCGTTGATCGTAAAGGAGTTGTGTTCCATCACGACAACGCGAGAC
HSal(Mariner_Tbel)     GGACAGTTTGAACGAATCCATCATCCAGAAACG------TCCAGAGCTCGTTAATCGTAAAGGAGTTGTGTTCCATCACGACAACGCGAGAC
PBa(Mariner_Tbel)      GGACAGTTTGAACGAATCCATCATCCAGAAACG------TCCAGAGCTCGTTAATCGTAAAGGAGTTGTGTTCCATCACGACAACGCGAGAC
EEu(Mariner_Tbel)      GGACAGTTTGAACGAATCCATCATCCAGAAATG------TCCAGAGCTCGTTAATCGTAAAGGAGTTGTGTTCCATCATGACAACGCAAGAC
CFl_(AEAB01001421)     GGACAGTTTGAACGAATCTGTCATCCAGAAACG------TCCGGAGCTCGTCAATCGTAAAGGAGTTGTGTTCCATCACGACAACGCGAGAC
CFl_(AEAB01018477)     GGACAGTTTGAACGAATCTGTCATCCAGAAACG------TCCGGAGCTCGTCAATCGTAAAGGAGTTGTGTTCCATCACGACAACGCGAGAC
SIn_(AEAQ01009575)     GGACAATTTAAACAAATCCATCATCCAGAAACGTTAATATCCAGAACTCGTTAACCATAAAGAAGTTGTGTTCCATCACGACAACGCAAGAC
SIn_(AEAQ01010279)     GGACAGTTTTAACGAATCTATCACCTAGAAATG------TATA-AATTCATTAATCGTAAAGGAGTTGTGTTCCATCACGACAACGCGTGAC
MRo_(AFJA01006902)     AGAAAGTTTGAAAGAATCAATTGAACAAAAACG------CCCAGAACTAGCCAACCGTAAAGGAGTTGTGTTCCAACACGATAATGCGAGAC
MRo_(AFJA01006736)     AGAGAGTTTGAAAGAATCAATTGAACAAAAACG------CCCAGAACTGGCCAATCGTAAAGGATTTATGTTCCACCCCGATAATGCGAGAC
Mariner-22_HSal        GGATAAATTGAATGCAGCGATCCACGAGAAGCG------TCCAGAATTGGTGAATCGTAGAGGCGTCGTCTTTCACCATGACAACGCTAGGC
Mariner-35_HSal        GGACTGTTTGAAGGCAGCAATCGATGAGAAGCG------TCCAGAATTGAGCAATCGTTATGGTGTCATATTCCACCAAGACAACGCTAGAC
Mariner1_BT            AGACCAACTGAAAGCAGCACTCGACGAAAAGCG------TCCGGAATTAGTCAACAGAAAACGCATAATCTTCCATCAGGATAACGCAAGAC
Mariner-5_ACe          AGACGAATTGAAGACAGCAATTGAACAAAAACG------TCCAGAAATAGCGAATCGGAAGGGCGTCGTGTTTCATCAGGACAATGCGCGGC
Mariner-1_DF           ATCCAATTTGGCGGAAAAAATTAAAGAAAAGGA------GCCGGCACTAGCTAATCGCAAGGGTATAGTCTTTCACCATGACAACGCTAGGC
Mariner-6_CFl          GGATAAATTGAAGATAGAAATCGCCAAAAAGTG------TCCGGAATTGATCAATAGGAAAGGCGTCGTTTTTCATCATGATAACGCCAGAC
Mariner-16_HSal        GGCCAAATTAAAGCGAGCAATCGATCAGAAGCG------GCCCGAATTGGCGAACAGAAAGGGCGTTGTGTTCCACCAGGACAACGCTAGAC
Mariner-47_HSal        GACGAAATTGAACCAAGCAATCCGCACAAAACG------TCCAGAATTGGCAAATCGCAAGGGTGTCGTCTTCCACCACGACAACGCCAGAC
Mariner-45_HSal        AGCGCGATTGCACCTAGCAATTCAAAAGAAACG------GCCGGAACTGGTCAACAGAAAGGGTGTCGTATACCACGCTGACAACGCCAGAC
AEc(Mariner-8_SIn)     GGATAGATTACAGGAAGCGATTAAGGAAAAACG------ACCAGAATTGATTAACAGAAAAGGTGTTGTCTTCCATCATGACAACGCCAGAC
Mariner-8_SIn          GGATAGATTACAGGAAGCGATTAAGGAAAAACG------ACCAGAATTGATCAACAGAAAAGGTGTCGTCTTCCATCATGACAACGCCAGAC
Mariner-16_AEc         GGAGAGATTACAACAAGCAATTGAGAGGAAGCG------GCCAGAATTAATCAATAGGAGGGGTGTCGTCTTCCATCACGACAACGCTCGAC
Mariner-1_DEl          GGACCGCTTGAAGGCAGCACTCATGCAGAAGAG------GCCATCTTTGATCAACAGAGGCCGAATTGTCTTCCATCAGGACAACGCCAGGC
Mariner-46_HSal        GACCAGATTGAAGCAGGCGATCGACGAGAAGCG------GCCAGAATTGGCCAATAGGAAGGGTGTTGTGTTCCATCAAGACAATGCCAGGC
Mariner-2_DEl          GGACCGTCTGAAGGAAGCAATTGCCCAGAAGCG------CCCCGCTTTGGCCAATAGGAAAGGAATTGTTTTCCATCAGGACAACGCTAGAC
Mariner-2_DEr          GGACCGCTTGAAGCAGGCGATCGACCAGAAGCG------TCCAGAATTGGCCAACAGGAAGGGTGTAGTGTTCCACCAGGACAACGCCAGAC
Mariner-28_SIn         GGACAAACTGAAGGAAGCCATTGCAGAAAAACG------CCCAGAATTGGTGAATAGAAAGGGTGTTTTATTCCATCAGGACAACGCCAGGC

                          1381
Mariner-24_SIn         CTCATACAAGTTTGGTAACTCGTCAAAAGCTTTTACAGCTTGGATGGGATATACTGCCACA--CCCACCATATTCTCCAGATCTGGCACCTT
SMAR7                  CTCATACAAGTTTGGTAACTCGCCAAAAGCTTTTACAGCTTGAATGGGATGTACTACCACA--CCCACCATATTCTCCAGATTTGGCACCTT
Mariner-11_HSal        CTCACACAAGTTTAATGACTCGCCAGAAGCTGTTAGCGCTTGGATGGGATTTGCTACCGCA--TCCACCATATTCACCTGATCTGGCACCAT
Mariner-1_AFl          CTCACACGAGTTTAATGACTCGCCAGAAGTTGTTACAGCTTGGATGGGAAGTGCTACCACA---CCACCATATTCGCCAGATCTGGCATCCT
Mariner-36_HSal        CACATACATCGTTGGTCACCCGCCAGAAATTGTTGCAGCTTGGGTGGGATGTGTTACCACA--CCCACCATATTCGCCTGACCTTGCACCAT
Mariner-23_HSal        CACACACAAGTTTGATGACTCGCGAAAAATTGTTGGAGCTTGGTTGGGATGTGTTGCCTCA--TCCACCGTATTCGCCAGACCTTGCACCAT
PBa(Mariner-23_HSal)   CACATACAAGTTTGATGACTCGCAAAAAATTGTTGGAGCTTGGTTGGGATGTGTTGCCTCA--TCCACCATATTCGCCAGACATTGCACCAT
Mariner-42_HSal        CCCATACATCTTTAGCAACACGTACGAAATTATTGGAGCTTGGTTGGGAAGTGATGTCGCA--TCCTCCATATAGCCCCGACCTTGCACCAT
Mariner-13_ACe         CATACACATCTTTGGCCACGCGGCAAAAATTATTGGAGCTTGGTTGGGATGTGTTGCCACWTGTCCGCCATACTCACCTGATCTTGCACCCT
AEc_AEVX01012963       CATACACATCTTTGGCCACGCGGCAAAAATTATTGGAGCTTGGTTGGGATGTGTTGCCACA--CCCGCCATATTCACCTGATCTTGCACCCT
Mariner-2_HSal         CACACACATCTTTGGTCACTCGGCAAAAATTATTGGAGCTTGGTTGGGATGTGTTGCCACA--TCCACCATATAGTCCTGACCTTGCACCAT
AMe(FAMAR1)            CACACACATCTTTGGTCACTCGGCAAAAATTATTGGAGCTTGGTTGGGATGTTTTGCCACA--TCCACCATATAGTCCTGACCTTGCACCAT
FAMAR1                 CACACACATCTTTGGTCACTCGGCAAAAATTATTGGAGCTTGGTTGGGATGTTTTGCCACA--TCCACCATATAGTCCTGACCTTGCACCAT
Mariner-1_BTe          CCCACACATCTTTGGTCACTCGCCAAAAATTATTGGAACTAGGTTGGGATGTGTTGTCACA--TCCACCATATAGCCCTGACCTTGCGCCTT
MARINER_CA             CACACACATCTTTGATGACCCGGCAAAAACTGAGAGAGCTTGGCTGGGAAGTTTTGATGCA--TCCACCATATAGCCCTGACCTTGCACCAT
Mariner-1_ACe          CACACACAAGTTTGGTCACTCGGCAAAAATTGTTGCAACATGGTTGGGATGTGTTACCACA--TCCACCGTATAGCCCAGACCTTGCACCAT
LHu_ADOQ01001582       CACACACAAGTTTGGTCACTCGGCAAAAATTGTTTCAGCTTGGTTGGGATGTGTTGCCACA--TCCACCATAT----CAGACCTTGCGCCAT
LHu_ADOQ01008024       CACACACAAGTTTGGTCACTCGGCAAAAATCGTTTCAGCTTGGTTGGGATGTGTTGCCACA--TCCACTATATAGCCCAGACTTTGCGCCAT
Mariner_Tbel           CACACACAAGTTTGATCACCCGTCAAAAATTATTGGAGCTTGGATGGGATGTGTTGCCACA--CCCACCATACTCGCCTGACCTTGCGCCGT
HSal(Mariner_Tbel)     CACACACAAGTTTGATCACCCGTCAAAAATTATTGGAGCTTGGATGGGATGTGTTGCCACA--CCCACCATACTCGCCTGACCTTGCGCCGT
PBa(Mariner_Tbel)      CACACACAAGTTTGACCACCCGTCAAAAATTATTGGAGCTTGGATGGGATGTGTTGCCACA--CCCACCATACTCGCCTGACCTTGCGCCGT
EEu(Mariner_Tbel)      CACACACAAGTTTGATCACCTGTCAAAAATTATTGGAGCTTGGATGGGATGTGTTGCCACA--CCCACCATACTCGCCTGACCTTGCGCCGT
CFl_(AEAB01001421)     CACACACATCTTTGGCGACCCGTCAAAAATTATTAGAGCTTGGATGGGATGTGTTGCCCCA--CCCAGCATACTCGCCTGACCTTGCGCCGT
CFl_(AEAB01018477)     CACACACATCTTTGGCGACCCGTCAAAAATTATTAGAGCTTGGATGGGATGTGTTGCCCCA--CCCAGCATACTCGCCTGACCTTGCGCCGT
SIn_(AEAQ01009575)     CACACACAAATTTGATCAGCCGTCAAAAATTATTAGAGCTTAGATAAGATGTGTTGCCACA--CCCACCATACTCGCCTAATCTGACGCCGT
SIn_(AEAQ01010279)     CATACATAAGTTTGATCACCTGTCAAAAATTATTGGAGCTTGGATAAGATGTGTTGCCACA--CCCACCATACTCG-----CATTAAGCCGT
MRo_(AFJA01006902)     CACACACAAGTTTGGTCACCCGCCAAAAATTGTTGGAGCTTGGATGGGATGTGCTGCCACA--TCCACCTTATTCGTCTGACATTG------
MRo_(AFJA01006736)     CACAGACGAGTGTGGTCACCCGCCAAAAATTGCTGGAGCTTGGATGGGATGTGCTGCCATA--TCCACCTTATTCACTTGACCTTGCACCGT
Mariner-22_HSal        CGCACACATCTCTACAAACCCGGCAAAAATTATTGGCGCTAGGCTGGGATGTCTTACCACA--TCCTCCGTATTCTCCAGACATTGCACCTT
Mariner-35_HSal        CTCACGTATCTTTGGCAACCCGGCAAAAATTATTGCAGCTTGGCTGGGATGTCTTGCCTCA--TCCTCCGTATTCACCTGACCTGGCACCAT
Mariner1_BT            CGCATGTTTCTTTGATGACCAGGCAAAAACTGTTACAGCTTGGCTGGGAAGTTCTGATTCA--TCCGCCGTATTCACCAGACATTGCACCTT
Mariner-5_ACe          CTCATGTGTCTTTGATAACTCGACAAAAGTTGTTGGAGCTCGGTTGGGATGTTCTACCCCA--TCCACCATATTCACCAGACCTCGCGCCCT
Mariner-1_DF           CCCACACATCTTTGGTCACTCGGCAAAAATTAACGGAGCTGAATTGGGAAGTGCTACCACA--TCCACCTTATAGTCCGGACTTAGCACCTT
Mariner-6_CFl          CTCATGCAAGTTTGCAGACTCGCCAAAAATTATTGGCGTTTGGCTGGGATGTTTTGCCTCA--TCCACCGTATTCCCCCGACATTGCACCCT
Mariner-16_HSal        CACACGTTTCTCTGATGACCCGGCAAAAGTTATTGCGGCTTGGGTGGGATGTTTTGGTGCA--CCCACCGTATAGTCCGGACCTTGCACCTT
Mariner-47_HSal        CTCATACGTCATTGACCACTCGGAACAAATTGCTTCAGCTTGGCTGGGATGTTTTGCCTCA--TCCTCCGTATTCACCGGATCTTGCCCCTT
Mariner-45_HSal        CGCATACATCTTTAACGACCCGTCAGAAATTGAGAGAACTTGGCTGGGAGGTTTTAATGCA--TCCACCGTATAGCCCGGACCTGGCACCGT
AEc(Mariner-8_SIn)     CACACACATCTTTGATGACGCGCCAAAAAATTGAGGGAGCTTGGTTGGGAAGTTTTGATGCA--TCCACCGTATAGCCCGGACCTTGCACCGT
Mariner-8_SIn          CACACACATCTTTGATGACGCGCCAAAAAATTGAGGGAGCTTGGTTGGGAAGTTTTGATGCA--TCCACCGTATAGCCCGGACCTTGCACCGT
Mariner-16_AEc         CACACACATCTTTGATAACTCGGCAAAAATTGCGAGAGCTTGGCTGGGAAGTCTTAATGCA--TCCACCGTATAGTCCTGACATAGCACCCT
Mariner-1_DEl          CACACACATCTTTAGTGACGCGCCAGAAGCTCCGGGAGCTCGGATGGGAGGTTCTTTTGCA--TCCACCGTATAGTCCGGATCTCGCACCAA
Mariner-46_HSal        CGCACACATCTTTGACGACGCGCCAGAAGCTCCGAGAGCTTGGATGGGAGGTTTATATCGCA--CCCGCCGTATAGCCCAGACCTGGCTCCAA
Mariner-2_DEl          CACACACGTCAATAGCGACTCGCCAGAAGCTCCGAGAGCTTGGATGGGAGGTTCTTTTGCA--TCCACCTTATAGCCCGGACATCGCACCAA
Mariner-2_DEr          CACACACTTCGTTGATGACTCGTCAGAAGCTACGGGAGCTCGGATGGGAGGTTTTATCGCA--TCCACCATATAGCCCGGACATAGCGCCAA
Mariner-28_SIn         CTCATGTTTCTTTAACCACCCGG-AAAAAATATTGCAACTTGGCTGGAATATTCTATCGCA--CCCAGCATATTCACCAGACATTGCTCCTT
```

```
                      1473
Mariner-24_SIn        CTGATTATTATTTG--TTCCGGTCTCTACAAAATTTTTTAGACGGTAAAACCTTTAC----TTCAAATGAGGAAGTCAAAAATCATCTGGAT
SMAR7                 CGGACTATTACTTG--TTCCGGTCTCTGCAAAGTTTTTTGGACGGGAAGACCTTCAC----TTCAAATCAGGACGTCAAATATCACTTGGAC
Mariner-11_HSal       CCGATTATCACTTG--TTTCGGTCACTGCAAAATTCTTTGGATGGTAAAAATTTCGA----TTCAAATGAAGGTGTCAAAAACCACTTAATT
Mariner-1_AFl         CGGATTACCATCTG--TTTCGATCTTTACAGAATTCTTTAGATGGCAAAACATTTAC----TTCAAATGAGGATGTCAAAAACCACTTGGAT
Mariner-36_HSal       CAGATTACCATTTA--TTTCGTTCCTTGCAAAATTCCTTGAAGGGAAAAAAATTCGA----TTCTGATGAGGCTATCAAACGGTACTTGGAT
Mariner-23_HSal       CAGACTACCATTTG--TTTCGGTCCTTGCAAAACTCCTTGAGTGGAAAAACGTTCGC----TTCAGATGACGCTATCGAAAGGCACTTGGTT
PBa(Mariner-23_HSal)  CAGACTACCATTTG--TTTCGATCCTTGCAAAACTCCTTGAGTGGTAAAACGTTCGC----TTCAGATGACGCTATCAAAAGACATTTGGTT
Mariner-42_HSal       CGGATTATCATTTA--TTTCGAAGTTTACAAAACTTTTTAAACGGTAAAAATTTCAA----CAACAACGATGATCTCAAATCGCACTTGGTT
Mariner-13_ACe        CGGACTACCATTTA--TTTCGATCATTGCAAAACTCTTTGAATGGAAAAACTTTCAC----CAATGATGATGATGTCAAATCGCACTTAGTT
AEc_AEVX01012963      CGGACTATCATTTA--TTTCGATCATTGCAAAACTCTTTGAATGGAAAAACTTTCAC----CAATGATGATGGTGTTAAATCGCACTTAGTT
Mariner-2_HSal        CAGATTATTATTTA--TTTCGTTCTTTGCAAAACTTCTTGAATGGTAAAAATTTCAA----TAATGATGATGATGTCAAATCGCACTTGGTT
AMe(FAMAR1)           CTGATTACTTTTTG--TTTCGATCTTTACAAAACTCCTTGAATGGTAAAAATTTCAA----TAATGATGATGATATCAAATCGTACCTGATT
FAMAR1                CTGATTACTTTTTA--TTTCGATCTTTACAAAACTCCTTGAATGGTAAAAATTTCAA----TAATGATGATGATATGTCAAATCGTACCTGATT
Mariner-1_BTe         CAGATTACCATTTA--TTTCGTTCCATGCAGAACTCCTTAAATGGTAAAATTTTTAA----TGACGCTGATGATGTAAAATCACATTTAATT
MARINER_CA            CAGACTACCATTTA--TTTCGATCTTTGCAGAACTCCTTAAATGGTAAAACTTTCGG----CAATGATGAGGCTATAAAATCGCACTTGGTT
Mariner-1_ACe         CAGACTTCCATCTC--TTTCGCTCCTTGCAAAACTCCTTGAATGGTAAAACGTTTGC----TTCTGAAGACCTCATCAAACAGCACCTAGAC
LHu_ADOQ01001582      CAGACTTCCATTTA--TTTCGCTCTTTGCAAAACTCTTTGAATGGTAAAACGTTTGC----TTCTGAAGACCTCATCAAACAGCACCTTGAC
LHu_ADOQ01008024      CAGACTTCCATTTA--TTTCGCTCTTTGCAAAACTCTTTGAATGGTAAAACGTTTGC----TTCTGAAGACCTCATCAAACAGCACCTTGAC
Mariner_Tbel          CAGACTTCCACTTG--TTTCGCTCTCTTCAAAACTCCTTGCGTGGTATAACGTTCAA----TTCAGATGAGGCTGTAAATCAGCACCTTGTT
HSal(Mariner_Tbel)    CAGACTTCCACTTG--TTTCGCTCTCTTCAAAACTCCTTGCGTGGTATAACGTTAAA----TTCAGATGAGGCTGTAAATCAGCACCTTGTT
PBa(Mariner_Tbel)     CAGACTTCCACTTG--TTTCGCTCTCTTCAAAACTCCTTGCATGGTATAACGTTTAA----TTCAGATGAGGCTGTAAATCAGCACCTTGTT
EEu(Mariner_Tbel)     CAGACTTCCACTTG--TTTCACTCTCTTCAAAACTCCTTGCGTGGTATAATGTTCAA----TTCAGATGAGGCTGTAAATCAGCACCTTGTT
CFl_(AEAB01001421)    CAGACTTCCACTTG--TTTCGCTCTCTTCAAAACTCCTTGCGTGGAATAACATTTAA----TTCAGATGAGGCGGTAAATCAGCACTTTATT
CFl_(AEAB01018477)    CAGACTTCCACTTG--TTTCGCTCTCTTCAAAACTCCTTGCGTGGAATAACATTTAA----TTCAGATGAGGCGGTAAATCAGCACTTTATT
SIn_(AEAQ01009575)    CAGACTTGCACTTG--TTTCGCTCTCTTC-AAACTCCTTGCGTGATATAACATTCAA----TTCAAAT-----TATAAATAAACACCTTGTT
SIn_(AEAQ01010279)    CAGATTTTCACCTGATTTTCGCTCTTTTCAAAATTCCTTGCGTGATATAACGTTTAA----TGCAGGTAAAGCTGTTAATCAGTCCCTTGTT
MRo_(AFJA01006902)    --------CACCTG--TTTCAGGCATTACAGAACTCCCTCCGTGGTGTAACTTTTAA----TTCTGACGAGGCGGCACATCAGCACCTTGTT
MRo_(AFJA01006736)    CAGATTTTCACCTG--TTTCACGCTTTACAGAACTCTCTACGTGGTGTAACTTTTAA----TTCTAACGAAGCGGTGCATCAGCATCTTGTT
Mariner-22_HSal       CAGATTACCATCTG--TTTCGCTCCCTTCAAAATTCCTTGAATGGAAAGCACTTCGT----TTGTGAGGAAGCTGTCAAACGACACTTGGAA
Mariner-35_HSal       CAGACTTCCACTTA--TTCAGGTCTCTGCAGAATTCCCTCAATGGAAAAAATTTCAC----TTCTTTGGAGGCCTGCAAAAAGCATCTTGAG
Mariner1_BT           CGGATTTCCATTTA--TTTCGGTCTTTACAAAATTCTCTTAATGGAAAAAAATTTCAA----TTCCCTGGAAGACTGTAAAAGGCACCTGGAA
Mariner-5_ACe         CTGATTTCCACCTA--TTTCGATCATTACAAAATTCCCTTAATGGAAAAAGCTTCAA----CTCTTTGGTCGAGGTAAAAATCACTTAGAA
Mariner-1_DF          CAGATTACCATTTG--TTTCGCTCTCTTCAAAACTCTTTGAATGGTAAAAACTTTGA----TTCAGATGAGGCTGTCGAAAACCACTTGTCT
Mariner-6_CFl         CGGATTTTCATTTA--TTTCGGTCGCTACAAAACTCAATTAATGGTTTGAACTTTGC----TTCTTTGCAACTCCTCAAAAACTACTTGGAC
Mariner-16_HSal       CAGACTATCACTTG--TTCCGGGCGCTACAGAACAATTTGAATAATAAAACCTTCAG----TTCCCTGGACGTCCTCAAAAACCACTTGGAT
Mariner-47_HSal       CCGATTATCATTTA--TTCCGGTCGCTGCAGAACTCTATGAACGGGAAAACCTTCAA----GGATGAAGAGGCCGTTAAAAACCACCTGGAT
Mariner-45_HSal       CAGACTATCATTTG--TTTCGGTCTCTGCAGAACTCCTTGAATGGTGTTAAGTTAGT----TTCAAAAGAGGCCTGTGAAAACCACGTGTCA
AEc(Mariner-8_SIn)    CTGACTACCATTTG--TTTCGATCTCTGCAGAACTCTCTTGATGGAAAAACATTGGC----CGACAAAAGAGCTGCCGAAAATCACTTGAAA
Mariner-8_SIn         CTGACTACCATTTG--TTTCGATCTTTGCAGAACTCTCTTGATGGAAAAACATTGGC----CGACAAAAGAGCTGCCGAAAATCACTTGAAA
Mariner-16_AEc        CAGACTATCATTTG--TTTAGGTCTCTGCAGAACTCTCTTAATGGTGTAAAGCTGGC----TTCAAAAGAGGCCTGTGAAAATCACTTGAAG
Mariner-1_DEl         GTGATTACCACCTG--TTTCTGTCCATGGCGAACGCGCTTGGTAGTCTGAAGTTGGC----CACAAGAGAGTCCTGTGAAAATTGGCTCTCC
Mariner-46_HSal       GCGATTATCACCTT--TTCAAGCATTTGCAAAATTTTCTTGATGGTACAAAACTGGC----CTCAAGAGAGGCTTGTGAAAATGAGCTGGTC
Mariner-2_DEl         GTTACTACCACCTT--TTCCTATCAATGTCGAACGATTTGGATAGTGTAAAATTAGC----TACAAGAGAGGATTGTGAAAACCGACTGGCT
Mariner-2_DEr         GTGATTACCACCTG--TTCCTGTCCATGGCGAATGCCCTTGGTGGTGTAAAGTTGAA----CTCAAAAGAGGCTTGTGAAAAGTGGCTGTCC
Mariner-28_SIn        CGGATTACTACTTG--TTCCGATCCTTACAGAACTTCCTTAATAGTAAGGCGTTTCACTTTTTCTTTGGAGGACTGCAAAAACCACCTTCAA

                      1565
Mariner-24_SIn        CAGTTTTTTGCCAGCAAAGAACAAAAATTTTATGAGCGTGGAATCATGCTACTGCCA-------------------------GAAAGATGGC
SMAR7                 CAGTTTTTTGCCAGCAAAGATCAGAAGTTTTATGAGCGTGGAATCAATCTCCTGCCC-------------------------GAAAGATGGC
Mariner-11_HSal       CAGTTTTTTGCCAATAAGGACCAGAAGTTCTACGAGCGTGGAATCATGTTATTACCT-------------------------CAAAGATGGC
Mariner-1_AFl         CAGTTTTTTGCAAACAAAGATCAGAAGTTCTACGAGCGTGGAATCATGCTACTATCT-------------------------AAAAGATGGC
Mariner-36_HSal       CAGTTTTTCGCCGATAAGGACCAGAAGTTCTATGAGGCCGGAATCATGAAGCTACCA-------------------------AACAGATGGC
Mariner-23_HSal       CAGTCTTCGCTAATACAGATAGGAAGTTTTTCGAGAATGGGATCATGAAGCTTACC-------------------------GAAAGATGGC
PBa(Mariner-23_HSal)  CAGTTTTTTGATAATAAAGATAGAAAGTTTTTCGAGAATGGGATCATGAAGCTTACC-------------------------GAAAGATGGC
Mariner-42_HSal       GAGTTTTTTGCTGATAAGGACCAGCAGTTCTATGAGCGCGGAATCATGAAGCTACCA-------------------------GAAAGATGGA
Mariner-13_ACe        AGGTTTTTTGCCGATAAGGATCAGAAATTTTATGAACAAGGCATTATGAAGCTGCCA-------------------------GAAAAATGGC
AEc_AEVX01012963      AGATTTTTTGCCGATAAGGATCAGAAATTTTATGAACAAGGCATTATGAAGCTGCCA-------------------------GAAAAATGGC
Mariner-2_HSal        CAGTTTTTTGCCGATAAAAACCAGAAGTTTTATGAACGTGGAATTATGACGCTGCCT-------------------------GAAAGATGGC
AMe(FAMAR1)           CAGTTTTTTGCTAATAAAAACCAGAAGTTTTATGAACGTGGGATTATGATGCTGCCT-------------------------GAAAGATGGC
FAMAR1                CAGTTTTTTGCTAATAAAAACCAGAAGTTTTATGAACGTGGGATTATGATGCTGCCT-------------------------GAAAGATGGC
Mariner-1_BTe         CAGTTTTTTGCTGGCAAAAATCAGAAGTTTTATGAACATGGAATTATGACACTGCCT-------------------------GAAAGATGGC
MARINER_CA            CAGTTTTTTGCAGATAAAGGCCAGAAGTTCTATGAGCGTGGAATACAAAATTTGCCG-------------------------GGAAGATGGC
Mariner-1_ACe         AAGTTTCTCGCAGAGAAGGATGGGAAGTTTTATGAACGTGGTATTATGAAGTTGCCC-------------------------GGGAGATGGC
LHu_ADOQ01001582      AAGTTTCTCGCAGAGAAAGATAGGAAGGTTTATGAACGCGGTATTATGAAGTTGCCC-------------------------GAGAGATGGC
LHu_ADOQ01008024      AAGTTTCTCGCAGAGAAAGATAGGAAGTTTTATGAACGCGGTATTATGAAGTTGCCC-------------------------GAGAGATGGC
Mariner_Tbel          CAGTTTTTCGCCGATAAAGACAGGTCCTTCTATGAACGAGGAATTATGAAGCTCACT-------------------------GAAAGATGGC
HSal(Mariner_Tbel)    CAGTTTTTCGCCGATAAAGACAGGTCCTTCTATGAACGAGGAATTATGAAGCTCACT-------------------------GAAAGATGGC
PBa(Mariner_Tbel)     CAGTTTTTCGCCGATAAAGACAGGTCCTTCTATGAACGAGGAATTATGAAACTCACT-------------------------GAAAGATGGC
EEu(Mariner_Tbel)     CAGTTTTTCACCGATAAAGACAGGTCCTTCTATGAACGAGGAATTATGAAGCTCACT-------------------------GAAAGATGGC
CFl_(AEAB01001421)    CAGTTTTTCGCCAATAAAGACAGGTCCTTCTACGAACGGGGAATTATGAAGCTCACC-------------------------GAAAGATGGC
CFl_(AEAB01018477)    CAGTTCTCGCCAATAAAGACAGGTCCTTCTACGAACGGGGAATTATGAAGCTCACCGAAAGAGGGAATTATGAAGCTCACTGAAAGATGGC
SIn_(AEAQ01009575)    CAGTTTTTTGTTGATAAAGAGA-----TTCTATGAACGAGGAATTATGAAGCTCACT-------------------------GAAAGATGGC
SIn_(AEAQ01010279)    CTGTTTTTTGCCAATA--GATAGATCCCTCTATGAACGAAAAATTATGAAGCTTATT-------------------------AAAAGATAGC
MRo_(AFJA01006902)    CAGTTTTT----------------------------------------------------------------------------------
MRo_(AFJA01006736)    CAGTTTTTTGCCAATAAAGACAACTCTTTCTTCGAACGAGGGATCATGAAGCTACCC-------------------------GAAAGATGAC
Mariner-22_HSal       CAGTTTTTTGCCCAGAAAGACAGGAAGTTCTTTGAACGAGGAATTATGAAGCTGCCC-------------------------GAAAGATGGC
Mariner-35_HSal       CAGTTTTTCGCCCAGAAAACTGGGAAGTTCTGGGAGGATGGAATCTTTAATTTGCCT-------------------------GAAAGATGGC
Mariner1_BT           CAGTTCTTTGCTCAAAAAGATAAAAAGTTTTGGGAAGATGGAATTATGAAGTTGCCT-------------------------GAAAAATGGC
Mariner-5_ACe         AAGTTTTTCGCTGAAAAACCTGAGAGATTCTGGAAGGACGGAATTTTCAAGTTGCCT-------------------------GAAAGATGGA
Mariner-1_DF          CGATTCTTTGCTGGAAAAGACCAAAAGTTCTTCGAGCGCGGAATTATGCAGCTGCCC-------------------------GAGAGATGGT
Mariner-6_CFl         AGCTTCTTCGCCGAAAAAACCCAAGACTTCTATGAGAGAGGAATTATGAAATTACCC-------------------------GAAAGATGGA
Mariner-16_HSal       GACTTCTTTGCCCAAAGATCTCAGGACTTCTATGAAAGAGGGATAATGAAGCTCGTG-------------------------GAGAGATGGC
Mariner-47_HSal       TGGTTTTTCGCCGATAAGCCCCAGACGTTTTACGAACGCGGCATAATGAAGTTGGTG-------------------------GAAAGATGGC
Mariner-45_HSal       CAATTTTTTGACCAGAAACCACAGAAGTTCTATAGCGACGGGATTATGGTGTTGCCG-------------------------GAAAAATGGC
AEc(Mariner-8_SIn)    AAGTTTTTCGCCGATAAACCACAAAAGTTCTACACGGATGGAATTATGAAATTACCC-------------------------GAAAAATGGC
Mariner-8_SIn         AAGTTTTTCGCCGATAAACCACAGAAGTTCTACACGGATGGAATTATGAAATTACCC-------------------------GAAAAATGGC
Mariner-16_AEc        CAGTTTTTCGACCAGAAACCACAGAAGTTCTATCGCGATGGGATTATGGCTCTGCCC-------------------------CAAAAATGGC
Mariner-1_DEl         GAGTTTTTTGCCAATAGGGAAGCGAGCTTCTATGAGAGGGGCATTATGAAGTTGGCA-------------------------TCTCGTTGGG
Mariner-46_HSal       AAGTTTTTTACCAATAGGGACGAGGACTTCTTCAATCGCGGAATTATGAAATTGCCT-------------------------TCAAAATGGA
Mariner-2_DEl         CAGTTTTTTGCAAATAAGGACGAAGGATTTTATGAGAGGGGTATCATGAAATTACCA-------------------------TCAAAATGGC
Mariner-2_DEr         GAGTTCTTCGCAAATAAGGAGGGGGGCTTCTACGAGGGAGGTATTATGAAGTTGCCG-------------------------TCTAGATGGA
Mariner-28_SIn        CAGTTTTTCGCTGAGAAAACGCCCGAGTTCTACGCGCGAGGAATTATGCAGCTGCCC-------------------------GAAAGATGGC
```

```
                  1657
Mariner-24_SIn        AAAAGGTATTGGACCA-----------------------GAATGGACAATATATAATTCAATAAAATATTTATTCA-----CTATA-AGA--
SMAR7                 AAAAGGTATTGGACCA-----------------------AAATGGAGAATATATAATTTAATAAAATGTTTATACA-----CTCTA-AAA--
Mariner-11_HSal       AAAAGGTGTTAGAACA-----------------------AAATGGTCAATACTTAACAGAATAAAACTTTTCTTTC-----TATTA-AAA--
Mariner-1_AFl         AACATGTATTGGACCA-----------------------CAATGGTCAATATGTAATTAAATAAAATACATATATG-----CTATT-AAA--
Mariner-36_HSal       AAAAGGTCATTGAATT-----------------------AAACGGACAATATATTACAGAATAAAGTTTTTGCTTT-----GTATG-AAA--
Mariner-23_HSal       AGAAGGTCATCGAACA-----------------------AAAAGGGCAATATATCATTGATTAATGTTCATTCTTT-----GTATA-AAA--
PBa(Mariner-23_HSal)  AGAAGGTCATCGAACA-----------------------AAGTGGGCAATACATCATTGATTAATATCAATTCTTT-----GTGTA-AAA--
Mariner-42_HSal       AAAAGGTCATCGAACA-----------------------GGATGGAAAATATTTGACCGATTAAAGTTCATTTTTT-----GTATAGAAA--
Mariner-13_ACe        AAAAGATCATTGAT-------------------------ATAATGACAATATATCATCGAATAAAGTTATTTCGTTGTACAATACA-AAA--
AEc_AEVX01012963      AAAAGATCATTGATCA-----------------------TAATGGACAATATATCATTGAATAAAGTTATTTCGTT-----ATACA-AAA--
Mariner-2_HSal        AAAAGGTCATTGATAAAAATGGACAANACGTCACTGCATAAATGGACAATACATTACTGCATAAAGTTATTTCGTT-----CCATG-AAA--
AMe(FAMAR1)           AAAAGGTCATTGATCA-----------------------AAATGGGCAACACATTACAGAATAAAGTTATTTAGTT-----CCATG-AAA--
FAMAR1                AAAAGGTCATTGATCA-----------------------AAATGGGCAATACATTACAGAATAAAGTTATTTAGTT-----CCATG-AAA--
Mariner-1_BTe         AAAAGGTCATCGACAA-----------------------AAACGGACAATACCTAATTGAATAAAGTTATATTTTT-----AAGCA-AAA--
MARINER_CA            AAAAGGTTATCGAACA-----------------------AAATGGAAATTATATATTTGATTAAAGTTCATTCTAA-----GTTTTATTA--
Mariner-1_ACe         AGAAGGTCATCGAACA-----------------------AAACGGCCAATATATCATTGATTAATGTTCATTACGT-----ATATTAAAT--
LHu_ADOQ01001582      AAAAGGTCATCGAACA-----------------------AAACGGCCGATATAGTATTGATTAATGTTCATTACGT-----ATATTAAAT--
LHu_ADOQ01008024      AAAAGGTCATCGAACA-----------------------AAACGGCCAATATATTATTGATTAATGTTCATTACGT-----ATATTAAAT--
Mariner_Tbel          AAAAGGTTATCGAACA-----------------------AAACGGGCAATATATCATTGATTGATCTTTGTTCTTT-----ATATA-AAT--
HSal(Mariner_Tbel)    AAAAGGTCATCGAACA-----------------------AAACGGGCAATATATCATTGATTGATCTTTGTTCTTT-----ATATA-AAT--
PBa(Mariner_Tbel)     AAAAGGTTATCGAACA-----------------------AAACGGGCAATATATCATTGATTGATCTTTGTTCTTT-----ATATA-AAT--
EEu(Mariner_Tbel)     AAAAGGTTATCGAACA-----------------------AAATGGGCAATATATCATTGATTGATCTTTGTTCTTT-----AT-----AT--
CFl_(AEAB01001421)    AAAAGGTCATCGAACA-----------------------AAACGGACAATATATCATTGATTGATCTTTGTTCTTC-----ATATA-AATAA
CFl_(AEAB01018477)    AAAAGGTCATCGAACA-----------------------AAACGGACAATATATCATTGATTGATCTTTGTTCTTC-----ATATA-AATAA
SIn_(AEAQ01009575)    AAAAGGTTATCGAACA-----------------------AAATGGGCAGTATATCATTGATTGATATTTGTTCTTT-----ATATA-AAT--
SIn_(AEAQ01010279)    AAAAGGT----------------------------------GGAACAAATATATCATTAATTAATCTTTGTTTTTT-----ATACA-AAT--
MRo_(AFJA01006902)    --------------------------------------------------------------------------------------------
MRo_(AFJA01006736)    AAAAGGTCATAAAACA-----------------------AAACGGGCAATACATCATAGACTAATT--------------------------
Mariner-22_HSal       AAAAGATAGTAGATAA-----------------------CAACGGCCAATACATAATTGATTAAAGTTATTACCTT-----CTATAAAAA--
Mariner-35_HSal       GAAAGGTTATCGAACA-----------------------AAACGGTGCATACATTGTTCATTAAAGGTATTTTTTA-----ATACG------
Mariner1_BT           AGAAGGTAGTGGAACA-----------------------AAACGGTGAATACGTTGTTCAATAAAGTTCTTGGTGA-----AAATG-AAA--
Mariner-5_ACe         GAAAGGTTGTAGAACA-----------------------GAATGGCACCTATATAATTTAATAAATGTATACTAAA-----ATTTA-AAT--
Mariner-1_DF          CATTAGTGGTCGAACA-----------------------AAACGGCCAATACATAATTGATTAATTATAAGTCCTG-----ATATA-AAT--
Mariner-6_CFl         AAAGTATTGTGCAAAA-----------------------TAATGGTGCATATATCACAGAATGAAATAACTTTTAA-----CAACA-AAA--
Mariner-16_HSal       AGAAAGTCATTGAACA-----------------------GAATGGAACATATTTAGTTGATTAAAATCGCTTTTTT-----TGCTA-AAT--
Mariner-47_HSal       TAGAGGTCATCGATAA-----------------------AGATGGCCAATATATAATTGATTAAAATTTATCTTGT-----GTATC-AAA--
Mariner-45_HSal       AGAAGGTCATCGAACA-----------------------AAATGGTGCATATGTGGTTTCATAATGTTATTTTCAA-----ATAAC-AAT--
AEc(Mariner-8_SIn)    AAAAGGTTATAGATAA-----------------------TAATGGCCAATATATACTTGATTAAAATTGATTTTCA-----ATAAA-GAA--
Mariner-8_SIn         AAAAGGTTATAGATAA-----------------------TAATGGCCAATATATACTTGATTAAAATTGATTTTCA-----ATAAA-GAA--
Mariner-16_AEc        AAAATATTATTGAAAA-----------------------TAATGGAGCATATTTGGTTTAAATAAATTGATTTTAA-----TACTC-TAA--
Mariner-1_DEl         AACGCGTCATCGAACA-----------------------AAACGGCGCATATTTGACTTGAATCGCATTATTGTAA-----CCAAT-TTT--
Mariner-46_HSal       CAAAAGTTATCGAACA-----------------------AAACGGCGCATATTTGATCTAAATCCGATAATCCTAA-----CTTTG-TTA--
Mariner-2_DEl         AATCGATTGTAGAAAA-----------------------AAATGGTGCTTATTTGAAATAAATCGGTTAATTCTAA-----CC--A-TAA--
Mariner-2_DEr         AACAGATTATCGAACA-----------------------AAACGGCGCATATTTGAACTAAATCCGATCACTGTAA-----CACTT-TTT--
Mariner-28_SIn        AAAAGGTTATAAAAAA-----------------------CAACGGCCATTACATCACTGATTGAAGCTGTTTTTAA-----ATATG-AAT--

                  1749
Mariner-24_SIn        -------AAATCGTCTTTCATTTTCATAAAAAAAAAAAAACGAAATTACTTTCCGAACAACCTA
SMAR7                 -------AAATCGTGTTTCATTTTCAC----TAAAAAAACGAAATGACTTTCCGAACAACCTG
Mariner-11_HSal       -------ATATCGTCTTTCATTTTCAT---GCAAAAAAACCGAAATTACTTTCCGAACAACCCA
Mariner-1_AFl         -------AAAATGTGTTTCATTTTGTC-ATGAAAAAAAACGAAATTACTTTCCGAACAACCCA
Mariner-36_HSal       -------AAATCGCTTTTCATTTACAT-AGAAAAAAAAACGACATGACTTTCTTGCCAGCCTG
Mariner-23_HSal       -------TAAACGGTCTTGAAAATCAT---CGGAAAAAACGCAATGATTTTTTGAACAACCCA
PBa(Mariner-23_HSal)  -------TGATCGGTCTTGAAAATCAT---CGAAAAAAACGCAATGACTTTTTGAACAACCCA
Mariner-42_HSal       -------AAATTGAGTTTTATTTCACA---CTAAAAAACCGAAATTACTTTCTTGCCAACCCA
Mariner-13_ACe        -------AAATTGGCTTCAGCWTTCTT---TTAAAAAAACGCAAKTACTTTCTTGCCAACCCA
AEc_AEVX01012963      -------AAATTGGTTTCAGCTTTCTT---TTAAAAAAACGCAATTGCTTTCTTGCCAACCCA
Mariner-2_HSal        -------AAATTGTCTTTTATTTTCTT---AAAAAAATCCGCAATTACTTAGTTGCCAAACCA
AMe(FAMAR1)           -------AAATTGTCTTTGATTTTCTA---AAAAAAATCCGCAATTACTTAGTTGCCAACCCA
FAMAR1                -------AAATTG--TTTGATTTTCT----AAAAAAATCCGCAATTATTTAGTTGCCAGCCCA
Mariner-1_BTe         -------ATTTTGAATTTTCCTTCTTA---TTTAAAATACGCAATCACTTAGTTGCCAACCCA
MARINER_CA            -------AAAATGCATTTACTTTCTTT---TAAAAAATCCGCAATTACTTTTTGGGCAACCCA
Mariner-1_ACe         -------AAACACACCTGAAAATCAAA---TGAAAAAAACGCAATGACTTTTTGAACAACCCA
LHu_ADOQ01001582      -------AAACACACCTAAAAATCAAA---TGAAAAAAACGCAATGACTTTTTGAACAACCCA
LHu_ADOQ01008024      -------AAACACACCTGAAAATCAAA---TGAAAAAAACGCAATGACTTTTTGAACAACCCA
Mariner_Tbel          -------AAATGACCTTTGAAAAACAA--GAAAAAAATACGTCATGACTTTTCTGACAACCCA
HSal(Mariner_Tbel)    -------AAATGACCTTTGAAAAACAT--AGAAAGAATACGTCATGACTTTTCCGACAACCCA
PBa(Mariner_Tbel)     -------AAATGACCTTTGAAAAACAT--AGAAAAAATACGTCATGACTTTTCCGACAACCCA
EEu(Mariner_Tbel)     -------AAATGACCTTTGAAAAACA----AAAAAAATATGTCATGACTTTTCTGACAACCCA
CFl_(AEAB01001421)    CTTTGAAAAACTGACTTTGAAAAACAT--ACAAAAAATACGCAATGACTTTTTACTCAACCCA
CFl_(AEAB01018477)    CTTTGAAAAACTGACTTTGAAAAACAT--ACAAAAAATACGCAATGACTTTTTACTCAACCCA
SIn_(AEAQ01009575)    -------AAATGATCTTTGAAAAACAT--AGAAAAAATAGGTCATGACTT--CCGACAACCCA
SIn_(AEAQ01010279)    -------AAATAATTTTTGAAAAACAT--AGAAAAAAGACGTCATGACTTTTCCAACAACCCA
MRo_(AFJA01006902)    -------TAACCGACTTCGAAA------------------------------------------
MRo_(AFJA01006736)    ----------------------------------------------------------------
Mariner-22_HSal       -------TAATCATCGATCATTTATAA---GTAAAAAACCGCACGGACTTATTGGCCAACCCA
Mariner-35_HSal       -------AAAATGTCTTTGAATTTCAC---CTAAAAATACGAAATTACTTTTTACTCAACCCA
Mariner1_BT           -------AATGTGTCTTTTATTTTTAC----TTAAAAACCGAAGGAACTTTTTGGCCAACCCA
Mariner-5_ACe         -------CTTCTGCGTTTCAATTTTCC---TTCAAAATCGGCACGAACTTTCCGGACAACCCA
Mariner-1_DF          -------AAATCATCTTTGAAAATATT---GAAAAAAAACGACATGACTTTCTTGCCAACCCA
Mariner-6_CFl         -------TTTTTTCATTCGAATTTTGC---TTAAAAATCCGCACGGACTTTCCGGACAACCCA
Mariner-16_HSal       -------ATTTTGCGTTTGAAATTTAT---TCGATTTTCGCTACGGAATTATGCATAGACCCA
Mariner-47_HSal       -------AAATTGTGTTTTATTCTACT---CTAAAAAACCGAACGAACTTATTTGCCAACCCA
Mariner-45_HSal       -------AAATGTATTCTCAATTTTGA---CCTCCAAAGGCTCAATACTTTTTAGACAACCTA
AEc(Mariner-8_SIn)    -------AAATTTCATTCAAAATTTGA---ACAAAAAACCGCGGATATTTTTTAGATGACCCA
Mariner-8_SIn         -------AAATTTCATTCAAAAATTGA---ACAAAAAACCGCGGATACTTTTTAAATGACCCA
Mariner-16_AEc        -------GAAAAACACTTGAATTTTAC---TAAAAAACCGCCACGAACTTTTTCCCACACCCA
Mariner-1_DEl         -------ATGAACAATTGAAAATTCAA----TAAAAATACCGCAAGACTTTTTTGACAACCTA
Mariner-46_HSal       -------ATTTCATCGTCGAAATAAAG---AGAAAAAACGCTCAGAACTTTTTACTTCACCCA
Mariner-2_DEl         -------AAAAAAGCTTTGAATTTCAT---GTACAAATAATGGATTACTTTTTACTTGACCTT
Mariner-2_DEr         -------ATAAAGCATTGAATAAAGAG---CAAAAAAGCGGAAGGGAGATATTTGCCA-----
Mariner-28_SIn        -------AAATTTGCTCCAATTTTAAC---TAAAAATACGACATTATTTTTTCTTCCAACCCA
```
